# Occurrence and health risk assessment of trace and heavy metals in four wastewater treatment plants and their receiving surface waters in Gauteng Province, South Africa

**DOI:** 10.1007/s10661-026-15686-3

**Published:** 2026-07-20

**Authors:** Thabile Lukhele, Titus Alfred Makudali Msagati

**Affiliations:** Institute for Nanotechnology and Water Sustainability (iNanoWS), College of Science Engineering and Technology, University of South Africa (UNISA), Science Campus, Johannesburg, South Africa

**Keywords:** Wastewater treatment plants, Sludge, River water, Metals, Human health risk assessment

## Abstract

Wastewater treatment plants (WWTPs) receive high volumes of domestic and industrial effluents, which are endowed with varying levels of metallic elements. If not properly treated WWTPs effluents discharged into river systems contribute to their contamination. This work reports on the ubiquitous detection of metals (As, Cd, Co, Cr, Cu, Fe, Mn, Pb, Ni, Zn) in WWTPs influents, effluents, sludge and water from the receiving rivers located in the Gauteng Province in South Africa. In wastewater, calculated mean metal concentrations ranged between 0.132 and 4.914 mg L^−1^. Comparatively in the surface water, the concentrations ranged between 0.127 and 4.631 mg L^−1^. In effluents As, Cr and Pb exceeded the permissible values for safe river discharge whilst surface water concentrations for As, Cd, Co, Fe, Mn, Ni and Pb exceeded the threshold values for agricultural and domestic use. These presented possible non carcinogenic and carcinogenic risks to individuals exposed to surface water through ingestion and dermal contact. In activated and digested sludge metal concentrations ranged from 16.425 to 2466 mg kg^−1^ with Zn and Cd being the most and least abundant respectively. Human health risk assessments for workers and farmers handling sludge revealed probable non-carcinogenic and carcinogenic risks emanating from incidental sludge ingestion. The findings of this work highlight the indiscriminate distribution of metals in the environment and support the calls for the upgrade of WWTPs to increase their efficiency in contaminant removal.

**Supplementary Information:**

The online version contains supplementary material available at 10.1007/s10661-026-15686-3.

## Introduction

At a global scale, the pollution of water systems is on the rise due to growing industrialisation, urbanisation and intensive agriculture. Metals are amongst chemical contaminants with a wide distribution in the environment because their sources and uses are far more extensive than other contaminants (Rathi et al., 2021). While metals occur naturally in the Earth's crust, various anthropogenic activities also contribute to metal discharge into the environment (Ali et al., 2019). These sources include industrial, municipal, agricultural and WWTPs discharges as well as leaded fuels, mining, mineral refineries and landfill leachates (Masindi et al., 2021). Their environmental distribution is cause for concern because metals are non-biodegradable, so they remain in the environment for extended periods of time. In aquatic ecosystems, metal elements attach to suspended particulate matter and settle in the sediments. Depending on the physicochemical parameters, these may be released into the water column, where they become a secondary source of pollution posing a threat to both aquatic flora and fauna as well as terrestrial users of water (Moiseenko et al., 2013; Sibal & Espino, 2018). Although some metals are essential for human metabolism, most metals (for example As, Cd, Cr, Hg, Mn, Ni, Pb) are highly toxic even at trace levels. Because of their bio accumulative nature, even if taken at low concentrations, overtime they build up in body tissues and cause detrimental effects on human health (Mitra et al., 2022). Some metals are human carcinogens while others are classified as mutagens and or teratogens to both humans and animals (Ali et al., 2019).

WWTPs are considered as hotspots for organic and inorganic contaminants including metals. Conventional WWTPs have limited efficiencies to remove metals from their influents because they are not technically designed for their removal (Cantinho et al., 2016). When improperly treated WWTPs effluents are reused as grey water in irrigation or discharged into surrounding freshwater bodies they can act as point sources of metal contamination in agricultural fields and aquatic ecosystems (Rathi et al., 2021). Furthermore, sewage sludge produced during wastewater treatment contains substantial amounts of metallic elements. Similarly, if used in agriculture for soil conditioning or for other land applications these contribute to metal pollution (Urbaniak et al., 2024).

The Gauteng Province in South Africa (SA), is a highly populated, industrialised economic hub with extensive urbanisation and mining activities. Human population growth, industrialisation and informal settlements characterise this province (Statistics South Africa, 2024). However, there seems to be a paucity of studies monitoring the occurrence of metals in WWTPs. This work was aimed at monitoring the occurrence of metals in the influent, effluent and sludge in four WWTPs in the Gauteng Province of SA. Furthermore, the occurrence of heavy and trace metals in four river systems receiving discharge from these plants was determined. The human health risks associated with exposure to sludge and the surface water were estimated using several indices.

## Materials and methods

### Study area

Sludge and wastewater samples were collected from four fully functional WWTPs servicing the Johannesburg City in the Gauteng Province of South Africa. These are designated as WWTP-1, WWTP-2, WWTP-3, and WWTP-4 and some of their properties are presented in Table 1. Surface water samples were also collected from nearby rivers (designated as RS-1, RS-2, RS-3 and RS-4) into which the wastewater effluent is discharged. For each river two sampling points were selected, one before the WWTP (upstream) and another after the WWTP effluent discharge (downstream).

**Table 1:** Properties of the four WWTPs under study (DWS, 2022)

Property	WWTP-1	WWTP-2	WWTP-3	WWTP-4
GPS coordinates	−26.01018, 27.83676	−26.27174, 27.92722	−29.79545, 30.99552	−26.31903, 27.90000
System design capacity (ML/day)	55	150	405	240
Design capacity percentage utilisation	71	77	91	85
Catchment population equivalent	> 150 000	750 000	> 1.6 million	> 850 000
Receiving stream	Crocodile River	Harrington Spruit	Jukskei River	Klip River

## Sample collection and pre-treatment

### Collection of water and sludge samples

Sampling was done on three campaigns over the course of one week in July 2023. At each WWTP wastewater (influent and effluent), as well as sludge (activated and anaerobically digested) was collected. Activated sludge is waste sludge produced in the primary settling tank, passed aeration tank and the secondary clarifier. This sludge is channelled to a bioreactor for anaerobic digestion by mesophilic or thermophilic microorganisms. Anaerobically digested sludge constitutes a large proportion of water (> 95%) and less biosolids (Badza et al., 2020). Liquid activated sludge samples were collected at the digester inlet. Anaerobically digested sludge samples were collected from the digestor outlet. Grab water samples were collected from two points along each of the receiving river systems corresponding to the four WWTPs. At each sampling point three samples were collected into acid washed 1L polypropylene bottles. Wastewater and surface water physicochemical parameters including pH, total dissolved solids (TDS) and electrical conductivity (EC) were measured on site using a multi parameter meter (YSI 6 series Sonde Marion, Germany). Immediately after sampling samples were acidified to pH 2 with nitric acid (Supra pure, 65%), preserved on ice and transported to the lab for further analysis.

### Sludge and wastewater pre-treatment

In the laboratory sludge samples were dried in a freeze drier to constant weight, milled to a fine powder using a pestle and mortar and homogenized by passing through a steel test sieve (200-mesh). To measure physicochemical parameters in sludge, 2 g of dry sample was suspended in 10 mL deionized water and shaken at 150 rpm for 4 h. The suspension was allowed to settle overnight at room temperature. The liquid part was carefully decanted, filtered and used to measure pH and EC using a multimeter (Muzeza et al., 2026).

Prior to metal analysis wastewater and sludge samples were subjected to acid digestion to oxidize the organic matter, solubilize metals, and simplify the matrix following standard protocols (Hassan et al., 2007). WWTP influent and effluent samples were digested in a microwave (Sineo MDS-6G, Supplied by SepSci, Johannesburg South Africa). Firstly a 20 mL volume of wastewater was measured into microwave bombs and mixed with 4.5 mL of nitric acid and 1.5 mL of hydrochloric acid (37%). The mixture was digested at 220 ⁰C for 30 min using the manufacturers’ preloaded programme specific for wastewater. Sludge samples were digested on a hot plate using nitric acid and hydrogen peroxide. Briefly 0.5 g of dried sludge was placed in a 250 mL Erlenmeyer flask and mixed with 10 mL nitric acid. The slurry was mixed, covered with a watch glass and heated at 95 ⁰C with reflux for 15 min. This step was repeated until no brown fumes were released, indicating complete oxidation. Thereafter the solution was heated without boiling at 95 ⁰C for 2 h. In the next step the solution was cooled at room temperature and mixed with 2 mL water and 3 mL hydrogen peroxide (30%). This mixture was covered and heated gently until there was no effervescence (U.S. EPA, 1996). After digestion the samples were cooled at room temperature and filtered through qualitative filter paper to remove particulates. The filtrates were transferred into 100 mL volumetric flasks and quantitatively made up to volume with ultra-pure water produced in a Milli-Q system (Merck Millipore, NY, USA).

## Analytical methods

Metals were quantified in an inductively coupled plasma mass spectrometer (ICP-MS) (Perkin Elmer, Nexion 350D) after proper dilution. The ICP-MS is a versatile, sensitive and high throughput technique for elemental analysis in environmental samples. The metallic elements are decomposed into their atomic species which are then ionized at very high temperatures in an argon plasma. Charged ions are channelled into the mass spectrometer where they are quantified (Kamunda et al., 2016). In the current work the target metals were As, Cd, Co, Cr, Cu, Fe, Mn, Ni, Pb and Zn. A multi-element analytical standard (ICP IV) containing 23 metals at 1000 mg L^−1^(Perkin Elmer, South Africa) was used to prepare calibration standards. A set of 7 calibration standards in the range 0.1–1 mg L^−1^ were prepared in 1% HNO_3_ (v/v). Calibration curves were drawn for each metal and the correlation coefficient (r^2^) calculated. The calculated r^2^ values ranged between 0.989–0.999. For each sample analysis was performed in triplicate and the mean concentrations were calculated taking the dilution factors into consideration.

### Quality assurance and control

To minimize contamination, all sampling bottles, glass ware and consumables used during analysis were washed thoroughly using the following protocol: washing in anionic detergent, rinsing with tap water, rinsing with deionized water, soaking in nitric acid (5%) for 24 h, rinsing with deionized water and drying. Furthermore, analytical grade chemicals and reagents were. A calibration blank (1% HNO_3_) was analysed after every 10 samples to monitor contamination and instrument drift. Relative standard deviation (RSD) was used to measure the instrument precision (intra and interday) and RSD values less than 10% were accepted.

### Data analysis

Data was uploaded on Microsoft Excel statistical package for the computation of means and standard deviation. The t-test at (p = 0.05) was performed to determine statistical differences between the means of influent and effluent; activated and digested sludge in each WWTP as well the upstream and downstream means in each river system. The levels of metals removed during the wastewater treatment process in each WWTP were estimated from the percentage removal efficiency calculated using Eq. 1:

1 $$R=\frac{\left(Ci-Ce\right)}{Ci} \times 100\mathrm{\%}$$

where C_i_ (mg L^−1^) is the mean metal concentration in the influent and C_e_ (mg L^−1^) is the corresponding mean metal concentration in the effluent (Olujimi et al., 2016).

Furthermore, the metal concentrations in effluent samples were compared to the SA guidelines for effluent disposal into rivers as outlined in the South African Water Act of 1998. Metal concentrations in sludge were compared to SA guidelines for sludge intended for agricultural use. On the other hand, surface water samples were compared to SA guidelines for domestic, and irrigation water use.

## Human health risk assessment (HHRA)

In this work, possible human health risk assessments were performed for surface water and digested sludge.

### Human health risk assessment of surface water

Human health risk assessment linked to the ingestion and dermal contact of surface water was evaluated from different indices presented in Eqs. 2–11 (Bakare & Adeyinka, 2022).

#### Exposure assessment

As a first step to the HHRA, the Average Daily Dose (ADD) (mg kg^−1^ day^−1^) calculated using Eq. 2–4, was computed to quantitatively estimate the metal daily intake through water ingestion (ADD_ingest_) and or dermal contact (ADD_dermal_) (Mileti´c et al., 2023).

2 $$ADDingest= \frac{Cs\times\; EFw\times EDw \times IRingest}{BW \times AT}$$

3 $$ADDdermal= \frac{Cw\times\; EFw\times EDw \times SA \times ABS \times ET\times Kp}{BW \times AT } \times CF$$

4 ADD=ADDingest+ADDdermal

C_w_ (mg L^−1^) is the measured concentration of each metal; ED_w_ is the estimated exposure duration (70 years); EF_w_ is the estimated frequency of exposure (350 day year^−1^); IR is the ingestion rate (2 L day^−1^); SA is exposed skin surface (18,000 cm^2^), ABS is the dermal absorption factor (0.001); K_p_ (cm h^−1^) is the dermal permeability constant adopted from literature (values shown in Table S1); ET is the exposure time (0.2 h day^−1^); BW is average body weight (61.8 kg); AT is average lifespan for both carcinogens and non-carcinogens (25500 days); and CF is the unit conversion factor for water (0.001 L cm^−3^) (Bakare &Adeyinka 2022; Mollo et al., 2022; Moloi et al., 2020; Kamunda et al., 2016).

#### Assessment of the non-carcinogenic risk

Possible non-carcinogenic health effects that could emanate from exposure to metals were estimated from the Hazard Quotient (HQ) and the Hazard index (HI) (Eqs. 5–8). HQ is a quantitative estimate of metal toxicity after prolonged exposure to a specific non-carcinogenic metal. For each metal the HQ*dermal* and HQ*ingest* values are summed up to obtain the Total Hazard Quotient (THQ) which is a measure of carcinogenic risks for a particular metal through the two exposure routes. HI is a summation of the HQ values for each sample and estimates the combined toxicity of different metals via various routes of exposure. HQ and HI values of less than 1 imply no possible adverse health effects. On the contrary HQ and HI values greater than 1, imply that human beings may tolerate the metal concentrations ingested, but non-carcinogenic effects are possible (Mileti´c et al., 2023).

5 $$HQingest= \frac{ADDingest}{RfDingest }$$

6 $$HQdermal= \frac{ADDdermal}{RfDdermal}$$

7 THQ=HQingest+HQdermal

8 $$HI={\sum}_{i=1}^{n}HQi$$

The reference dose (RfD) is an estimated metal dose that an individual can be exposed to over an extended period of time without showing any adverse health effects. Oral reference dose (RfD_ingest_) and dermal reference dose (RfD_dermal_) values used in this study were adopted from literature and are presented in Table S1.

#### Assessment of carcinogenic risk

Possible carcinogenic effects arising from exposure to carcinogenic metals (As, Cd, Cr, Ni, Pb) were estimated with the Carcinogenic Risk factor (CR) for oral (CR_ingest_) and dermal (CR_dermal_) exposure pathways as well the Total Carcinogenic Risk Factor (TCR) and the Carcinogenic Risk Index (RI) calculated from Eqs. 9–11.

9 CR=ADD×CSF

10 TCR=CRingest+CRdermal

11 $$RI=\sum_{i=1}^{n}\left(ADD \times CSF\right)$$

CSF is the cancer slope factor for either oral (CSF_ingest_) or dermal (CSF_dermal_) exposure (Table S1). CR and TCR threshold values are set as follows: CR ≤ 1 × 10^–6^ imply negligible carcinogenic risks; CR ≤ 1 × 10^–5^ low risk; CR ≤ 5 × 10^–4^ medium risk; CR = 5 × 10^–4^ – 1 × 10^–3^ high risk; CR ≥ 1 × 10^–3^ extremely high risk (Mileti´c et al., 2023; Rathi et al., 2021).

### Human health risk assessment of sewage sludge

To assess the possible human health risks arising from the ingestion of metals present in sewage sludge the following indices (Eqs. 12–17) were calculated:

#### Exposure assessment

In addition to (ADD_ingest_) and (ADD_dermal_), ADDinhale (Eqs. 12–14) was calculated to estimate the levels of metal daily intake from exposure from sludge through ingestion, dermal contact and inhalation.

12 $$ADDingest= \frac{Cs\times EFs\times EDs \times IRingest}{BW \times AT } \times CF$$

13 $$ADDdermal= \frac{Cs\times EFs\times EDs \times SA \times AF \times ABS}{BW \times AT } \times CF$$

14 $$ADDinhale= \frac{Cs\times EFs\times EDs \times IRinhale}{PEF \times BW\times AT } \times CF$$

Cs (mg kg^−1^) is the concentration of each metal in sewage sludge; EF_S_ is the exposure frequency (260 days year^−1^); ED_s_ is the estimated exposure duration (30 years); IR_ingest_ is the rate of metal intake (100 mg day^−1^); SA is the skin surface area exposed to sludge (4850 cm^2^). ABS is the dermal absorption fraction for each metal (0.001). IR_inhale_ is the inhalation rate (20 m^−3^ day^−1^); PEF is the particulate emission factor (1.36 × 10^9^ m^3^ kg^−1)^ (Mileti´c et al., 2023; Rocha et al., 2025).

#### Assessment of the non-carcinogenic risk

HQ, THQ and HI were calculated to estimate the non-carcinogenic risks emanating from incidental ingestion (HQ_oral_), dermal contact (HQ_dermal_) and inhalation (HQ_inhale_) of sewage sludge (Eqs. 5, 6,8, 15–16).

15 $$HQinhale=\frac{ADDinhale}{RfDinhale}$$

16 THQ=HQingest+HQdermal+HQinhale

#### Assessment of the carcinogenic risk

Carcinogenic risks were estimated from CR, TCR and IR (Eqs. 9, 11, 17).

17 TCR=CRingest+CRdermal+CRinhale

## Results and discussion

### Physicochemical properties

Prior to quantifying the metal concentrations, wastewater, sludge and surface water samples were characterised to get a comprehensive understanding of their chemical and physical nature as well as ascertain their suitability for domestic or agricultural use and safe environmental disposal.

### Sludge

**Fig. 1:**
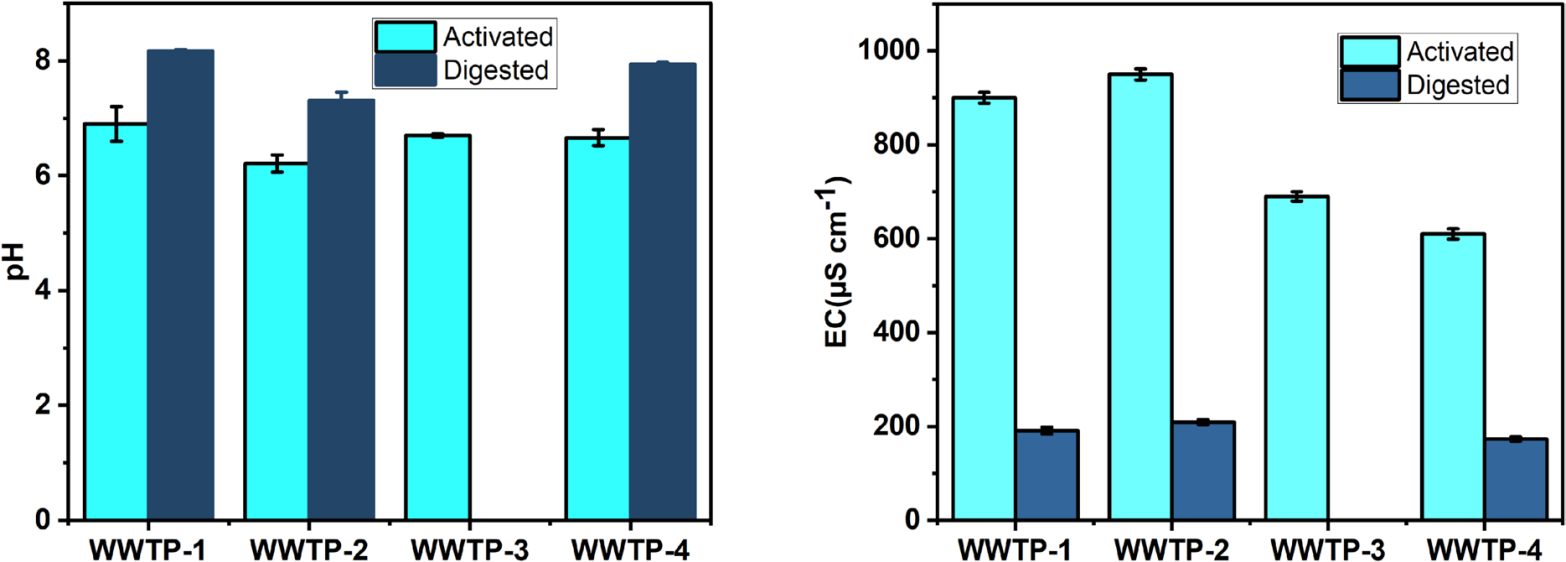
Physicochemical parameters of activated and digested sludge collected from four WWTPs. Each value is a mean of nine replicate samples and error bars represent the standard deviation

Activated and anaerobically digested sludge were characterised in terms of pH and EC (Fig. 1). For sewage sludge, the pH, EC and other parameters are influenced by the type of influent received as well as the wastewater treatment processes. These parameters are particularly important because they control the partitioning of metal ions between wastewater and sludge as well as determine the possible applications of sludge (Cantinho et al., 2016). For example, depending on the pH and rate of application sludge used in agriculture, can alter soil pH hence nutrient uptake and plant growth. For instance, at low soil pH (< 4.5) the availability of macronutrients such as N, P, K, Ca and Mg is restricted whilst at high soil pH (> 9.0) the availability of micronutrients (Fe, Mn, and B) may be compromised (Urbaniak et al., 2024). Additionally, sludge with high EC can increase soil salinity if applied to agricultural soils. High soil salinity causes oxidative stress and is toxic to plants hence it negatively affects plant growth and productivity (Snyman & Herselman, 2006). For the studied WWTPs activated sludge was slightly acidic with pH values ranging between 6.21 and 6.90. Comparatively, digested sludge pH values were significantly higher with values in the range 7.31—8.17. The activated sludge EC values ranged between 580 and 950 µS cm^−1^ compared to 173 and 210 µS cm^−1^ for digested sludge. The reduction in EC implies that some ionic species are removed during wastewater treatment processes. The digested sludge pH and EC values were compliant with the DWAF guidelines of (pH 8–9) and EC (200 mS m^−1^) recommended for sludge reserved for land applications (Snyman & Herselman, 2006).

### Wastewater

**Fig. 2:**
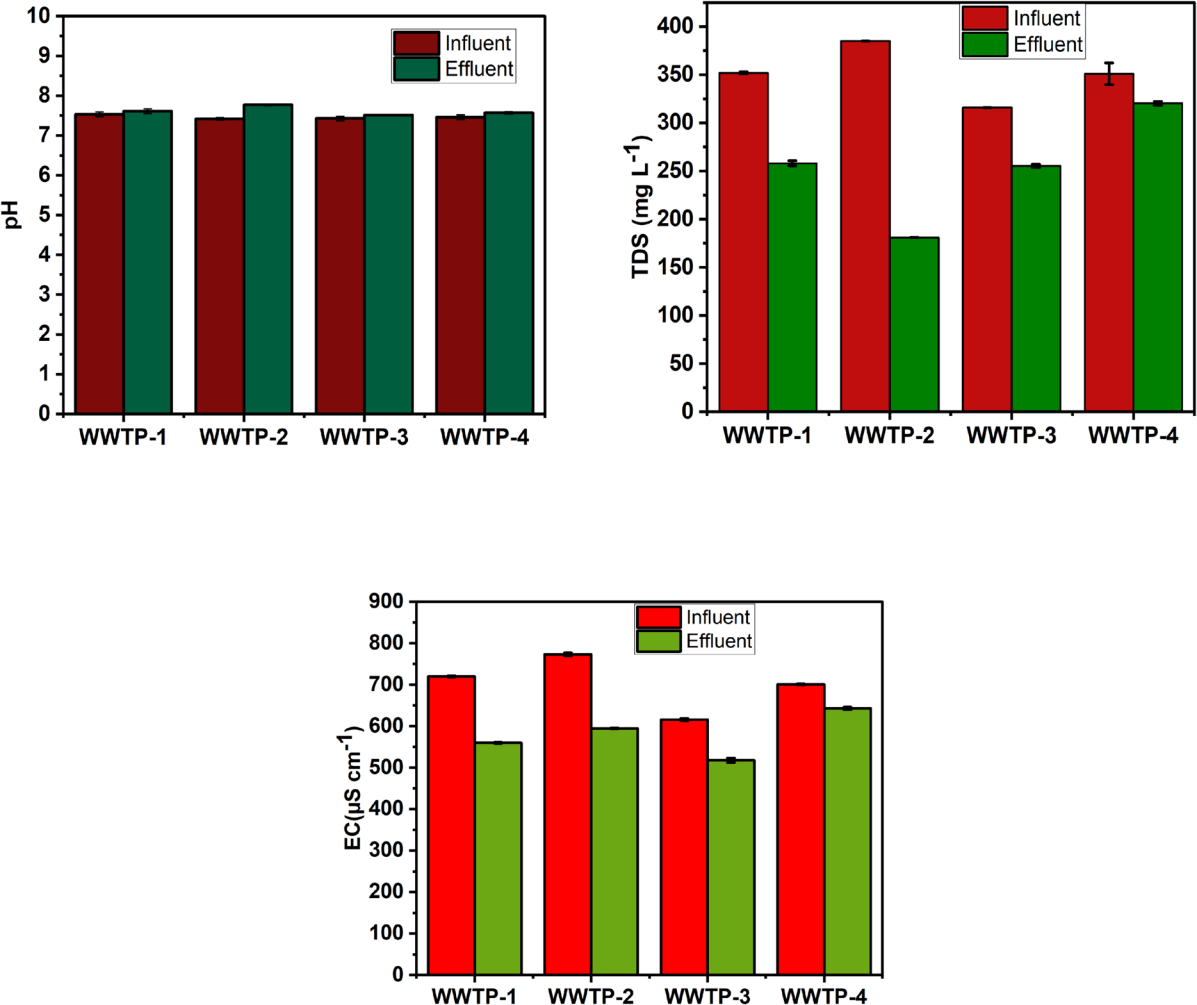
Physicochemical parameters of wastewater collected from four WWTPs. Each value is a mean of nine replicate samples and error bars represent the standard deviation

The pH, EC and TDS of WWTP influent and effluent were measured (Fig. 2). For all influents and effluents near neutral pH values in the range 7.42 to 7.57 (influent) and 7.46 to 7.77 (effluent) were measured. In all four WWTPs effluent pH values were significantly higher than influent values (p = 0.002, 0.0002, 0.008 and 0.0003 for WWTP-1, WWTP-2, WWTP-3 and WWTP-4 respectively). Chemicals applied at different stages of the wastewater treatment process are primarily responsible for the increase in pH. The TDS values reported for influent and effluent samples were in the range 316—385 mg L^−1^ (influent) and 181—320 mg L^−1^ (effluent). For WWTP-1, WWTP-3 and WWTP-4 the effluent TDS values were significantly lower than influent values (p < 0.05). For example, in WWTP-1 the mean TDS values decreased from 358 mg L^−1^ in the influent to 258 mg L^−1^ in the effluent. Electrical conductivity values were in the range 615.67—773.00 µS cm^−1^ (influent) and 517.67—643.00 µS cm^−1^ (effluent). Similarly effluent EC values were significantly lower (p < 0.05) than influent values in all four plants. The decrease in TDS and EC values implies that some ionic species dissolved in wastewater are removed during treatment. Different effluent physicochemical parameters are important factors to consider for safe disposal and re-use of WWTP effluents hence are regulated (National Water Act, 1998). For instance, EC is an indication of the levels of ionic and non-ionic species dissolved in the water. The effluent pH and EC values complied with the DWAF and WHO thresholds for pH (6—9) and EC (150 mS m^−1^) stipulated for safe environmental discharge of WWTPs effluents (Department of Water Affairs, 1999). Therefore, pH and EC were not limiting factors for possible reuse or river discharge of the effluents from the four WWTPs.

### Surface water

**Fig. 3:**
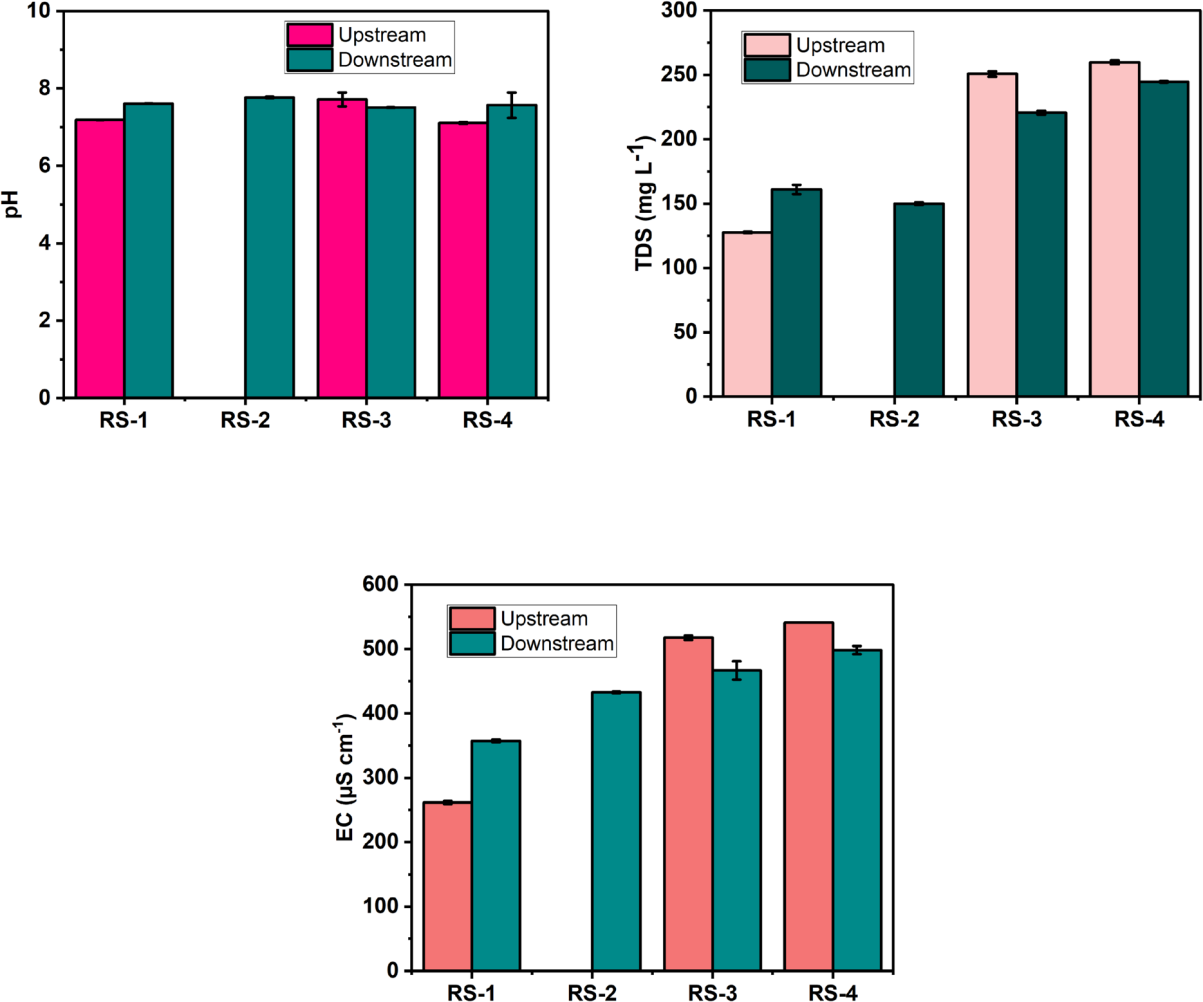
Physicochemical properties of surface water collected from four rivers receiving WWTP effluent discharge. Each value is a mean of nine replicate samples and error bars represent the standard deviation

For the surface water collected from rivers receiving treated effluents from the four WWTPs, water pH was near neutral ranging from 7.11 to 7.22 for upstream points and 7.14 to 7.80 for downstream points (Fig. 3). At RS-1 downstream pH values were significantly (p = 0.0001) higher than upstream values. Conversely for RS-3 the downstream pH values were significantly lower than upstream values (p = 0.015) whilst for RS-4 there was no significant difference between upstream and downstream values (p = 0.483). Upstream TDS values ranged between 127.67—259.67 mg L^−1^ compared to downstream values of 150—244 mg L^−1^. For RS-1 downstream values were significantly higher than upstream values (p = 0.004). On the contrary RS-3 and RS-4 downstream values are significantly lower than upstream values (p = 0.004, 0.002). EC values for the four river systems were in the range 261.67—541.00 µS cm^−1^ (upstream) and 357— 498.33 µS cm^−1^ (downstream). Compared to upstream values, downstream EC values were significantly higher in RS-1 (P = 0.0001), lower in RS-3 (p = 0.001) and not significantly different in RS-4 (p = 0.168). For RS-1 the downstream pH, TDS and EC values were significantly higher than the upstream values. These suggest that WWTP-1 effluents discharged into the river system possibly have an effect on the surface water pH, TDS and EC. Nonetheless for all four rivers surface water pH, and EC values complied with the DWAF and WHO guidelines for pH and EC in surface water (National Water Act, 1998).

## Heavy and trace metal analysis

### Metal concentrations in sewage sludge

Sewage sludge or WWTP sludge constitutes mainly the solids removed during the wastewater treatment processes. It is produced mainly during primary and secondary treatment stages. Depending on the influent composition and the treatment methods used, about 70% of metals present in the influent wastewater are transferred to sludge through physical, chemical and biological mechanisms (Fijalkowski et al., 2017). In this work substantial amounts of heavy and trace metals were detected in the activated and anaerobically digested sludge (Table 2). Zn was detected as the most abundant metal with the mean concentrations ranging between 1525.775 and 2466.036 mg kg^−1^. Other metals that were detected in abundance were Ni, Mn, Co and Cr. For instance, in WWTP-1, Zn was the most abundant metal with mean concentrations of 2466.036 mg kg^−1^ in activated sludge and 2085.274 mg kg^−1^ in digested sludge. The metal concentrations decreased in the order Zn > Ni > Mn > Cr > Fe > Cu > Pb > Co > Cd in activated sludge compared to Zn > Ni > Mn > Cu > Pb > Cr > Co > Fe > Cd in the treated sludge. Available literature indicates that Zn, Ni and Pb are some of the metals that are abundant in sewage sludge. For example, for Gauteng WWTPs activated sludge Pb (22–807 mg kg^−1^), Zn (560–2820 mg kg^−1^), Fe (8–24 g kg^−1^) was the most abundant and in anaerobically digested sludge the concentrations were Pb (38–1371 mg kg^−1^), Zn (710–10 400 g kg^−1^), Fe (21–109 g kg^−1^). Zn was the most abundant metal in 5 WWTPs located in Limpopo (951.25–1732.00 mg kg^−1^) (Shamuyarira & Gumbo, 2014) and those from the Eastern Cape (1600—4100 mg kg^−1^) (Morrison et al., 2004). During anaerobic digestion metals become concentrated as organic matter is lost (Badza et al., 2020; Moloi et al., 2020). When comparing the activated and treated sludge there were no clear trends in metal abundance. For some metals (Cu, Ni and Fe) there were significant differences between activated and digested sludge concentrations. For Pb in WWTP-1 and Co in WWTP-2 digested sludge concentrations were significantly higher than activated sludge. Otherwise for Co, Zn, Mn (WWTP-1); Pb, Cu (WWTP-2), as well as Zn (WWTP-4) the digested sludge concentrations were significantly lower than the activated sludge. Generally, the reported metal concentrations are comparatively higher than previously reported in different provinces including Limpopo (Shamuyarira & Gumbo 2014), Gauteng Eastern Cape (Agoro et al., 2020), (Morrison et al. 2004). However, compared to some WWTPs around Gauteng the concentrations are low (Badza et al., 2020).

**Table 2:** Metal concentrations (mg kg^−1^) in activated and anaerobically digested sludge collected from four WWTPs

Metal	WWTP-1		WWTP-2		WWTP-3	WWTP-4	
	activated	digested	activated	digested	activated	activated	digested
Cd	16.42 ± 2.99	16.49 ± 2.72	16.47 ± 3.39	29.47 ± 4.54	25.86 ± 3.14	21.78 ± 4.53	20.41 ± 4.59
Co	51.26 ± 18.89	101.34 ± 14.97	184.80 ± 19.78	471.88 ± 46.20	90.09 ± 38.67	481.75 ± 66.88	465.56 ± 91.98
Cr	312.11 ± 31.41	112.37 ± 33.71	372.61 ± 89.62	635.17 ± 58.42	995.51 ± 59.72	210.38 ± 36.57	210.96 ± 36.57
Cu	201.66 ± 36.21	168.05 ± 76.37	486.73 ± 35.58	48.21 ± 6.47	207.70 ± 30.24	23.77 ± 1.90	191.70 ± 24.4
Fe	266.56 ± 34.26	82.33 ± 8.12	161.00 ± 24.40	173.91 ± 15.58	41.99 ± 0.15	154.18 ± 11.33	306.99 ± 37.64
Mn	708.09 ± 29.46	182.95 ± 83.81	1075.19 ± 73.44	1596.26 ± 45.95	1699.15 ± 78.83	375.08 ± 9.88	846.02 ± 22.54
Ni	822.99 ± 31.38	472.74 ± 30.93	62.71 ± 2.35	590.26 ± 46.63	896.24 ± 42.46	626.42 ± 51.27	339.87 ± 34.02
Pb	97.02 ± 5.33	125.98 ± 8.90	151.79 ± 8.39	81.84 ± 2.36	188.03 ± 32.48	200.21 ± 60.78	205.12 ± 74.36
Zn	2466.04 ± 85.36	2083.27 ± 176.37	2302.18 ± 165.13	1525.77 ± 431.11	1543.28 ± 654.01	1329.94 ± 528.36	1133.06 ± 431.09

The safe and sustainable disposal or storage of sewage sludge has become a big issue. Commonly used methods such as land disposal and incineration are limited by the unavailability of land and high energy demands respectively. Consequently, sludge re-use and beneficiation has become necessary to circumvent these issues. However, proper monitoring is crucial to prevent environmental degradation from the various contaminants present in sludge (Badza et al., 2020). In SA, based on the concentrations of some potentially toxic metals (As, Cd, Cr, Cu, Pb, Hg, Ni, Zn) sewage sludge can be categorized into three classes: a (high quality), b (moderate quality), and c (worst quality). The pollutant subclass determines the permissible potential uses of sludge with Class a recommended for agricultural use whilst b and c have some restrictions (Table S2) (Snyman & Herselman, 2006). In WWTP-4 the concentrations of all 6 regulated metals (Cd, Cr, Cu, Pb, Ni, Zn) were within the permissible limits for pollutant class a. On the contrary in WWTP-1, WWTP-2, WWTP-3 the concentrations of Ni exceeded the permissible levels for class a and b (420 mg kg^−1^) downgrading it to class c (> 420 mg kg^−1^). Therefore, sewage sludge from these plants is less suitable for land applications.

### Metal concentrations in wastewater

The ten metals under study (As, Cd, Co, Cu, Cr, Fe, Mn, Ni, Pb and Zn) were detected in the influents and effluents of the four WWTPs located around Johannesburg in the Gauteng Province (Table 3). Generally, in all plants Fe, Mn, and Zn were the most abundant whilst Cd, Co, Cr, and Ni were the least abundant. In WWTP-1 samples the metal concentrations ranged between 0.133 (Cr) and 2.65 mg L^−1^ (Fe). Metal abundance decreased in the order Fe > Mn > Zn > Cu > As > Pb > Ni > Cd > Co > Cr in the influents compared to Fe > Zn > Mn > As > Pb > Cu > Co > Ni > Cd > Cr in the effluents. For WWTP-2 the measured metal concentrations ranged between 0.132 mg L^−1^ (Cr effluent) and 4.914 mg L^−1^ (Fe influent). The sequence of decreasing abundance was Fe > Mn > Zn > As > Cu > Ni > Co > Pb > Cd > Cr in influent compared to Fe > Mn > Zn > As > Cu > Co > Pb > Cr > Cd > Cr in effluents. In WWTP-3, which is the largest of the 4 WWTP’s, the influent metal concentrations were in the range 0.135—2.734 mg L^−1^ with Cr and Fe as the least and most abundant metals respectively. The order of decreasing abundance was Fe > Zn > Mn > Cu > As > Ni > Pb > Cd > Co > Cr. The effluent metal concentrations ranged from 0.137 to 0.846 mg L^−1^ in the sequence Fe > Zn > Mn > As > Cu > Pb > Ni > Cd > Co > Cr. In WWTP-4 the influent metal concentrations decreased in the order; Fe > Mn > As > Zn > Ni > Cu > Pb > Co > Cr > Cd with values in the range 0.144 −1.66 mg L^−1^. In the effluent the concentrations ranged between 0.131 to 0.436 mg L^−1^ and the order of decreasing abundance was Zn > Mn > As > Zn > Ni > Cu > Pb > Co > Cr > Cd. The occurrence of a variety of metals in the influents is indicative of the diversified industrial, agricultural and domestic activities within the catchments of the four WWTPs (Hoogendijk et al., 2023). Furthermore, the observed trend in metal abundance could be explained on the basis that metals such Fe, Zn and Cu have ubiquitous sources including natural sources, as well as domestic and industrial waste. On the contrary most of the less abundant metals including Cd are highly toxic and therefore are subject to stringent regulatory controls which restricts their use (Jaishankar et al., 2014). In Table S3 the concentrations of metals from the 4 WWTPs under study are compared with those of other local WWTPs from different Provinces. There is notably a wide variation in the metal concentrations amongst the different WWTPs. The highest concentrations for As, Fe and Mn were reported for the Gauteng WWTPs.

**Table 3:** Metal concentrations (mg L^−1^) in wastewater samples collected from four WWTPs

Metal	WWTP-1		WWTP-2		WWTP-3		WWTP-4		Permissible value
	influent	effluent	influent	effluent	influent	effluent	influent	effluent	
As	0.368 ± 0.047	0.363 ± 0.045	0.374 ± 0.058	0.391 ± 0.069	0.372 ± 0.053	0.380 ± 0.046	0.401 ± 0.068	0.390 ± 0.075	0.02
Cd	0.140 ± 0.007	0.138 ± 0.004	0.140 ± 0.008	0.139 ± 0.004	0.140 ± 0.007	0.140 ± 0.003	0.144 ± 0.003	0.138 ± 0.005	0.005
Co	0.136 ± 0.013	0.145 ± 0.029	0.192 ± 0.083	0.188 ± 0.063	0.138 ± 0.015	0.138 ± 0.005	0.153 ± 0.021	0.137 ± 0.011	N/A
Cr	0.133 ± 0.015	0.135 ± 0.017	0.137 ± 0.019	0.132 ± 0.011	0.136 ± 0.018	0.138 ± 0.012	0.150 ± 0.030	0.132 ± 0.011	0.05
Cu	0.405 ± 0.033	0.178 ± 0.065	0.301 ± 0.119	0.279 ± 0.070	0.386 ± 0.183	0.310 ± 0.135	0.191 ± 0.075	0.179 ± 0.058	0.01
Fe	2.659 ± 0.962	0.680 ± 0.702	4.813 ± 0.672	4.914 ± 0.019	2.734 ± 0.308	0.846 ± 0.523	1.668 ± 0.032	0.374 ± 0.272	0.3
Mn	0.674 ± 0.054	0.391 ± 0.032	1.512 ± 0.675	2.014 ± 0.923	0.581 ± 0.028	0.605 ± 0.334	1.470 ± 0.100	0.145 ± 0.027	0.1
Ni	0.155 ± 0.037	0.142 ± 0.023	0.231 ± 0.131	0.246 ± 0.126	0.195 ± 0.082	0.182 ± 0.042	0.192 ± 0.055	0.184 ± 0.066	N/A
Pb	0.189 ± 0.019	0.193 ± 0.024	0.191 ± 0.022	0.188 ± 0.014	0.186 ± 0.016	0.190 ± 0.010	0.183 ± 0.010	0.185 ± 0.012	0.01
Zn	0.625 ± 0.193	0.458 ± 0.011	0.588 ± 0.006	1.100 ± 0.240	0.608 ± 0.132	0.715 ± 0.140	0.366 ± 0.010	0.437 ± 0.057	0.1

*Guidelines set by DWA (National Water Act, 1998)

#### Metal removal efficiencies

**Fig. 4:**
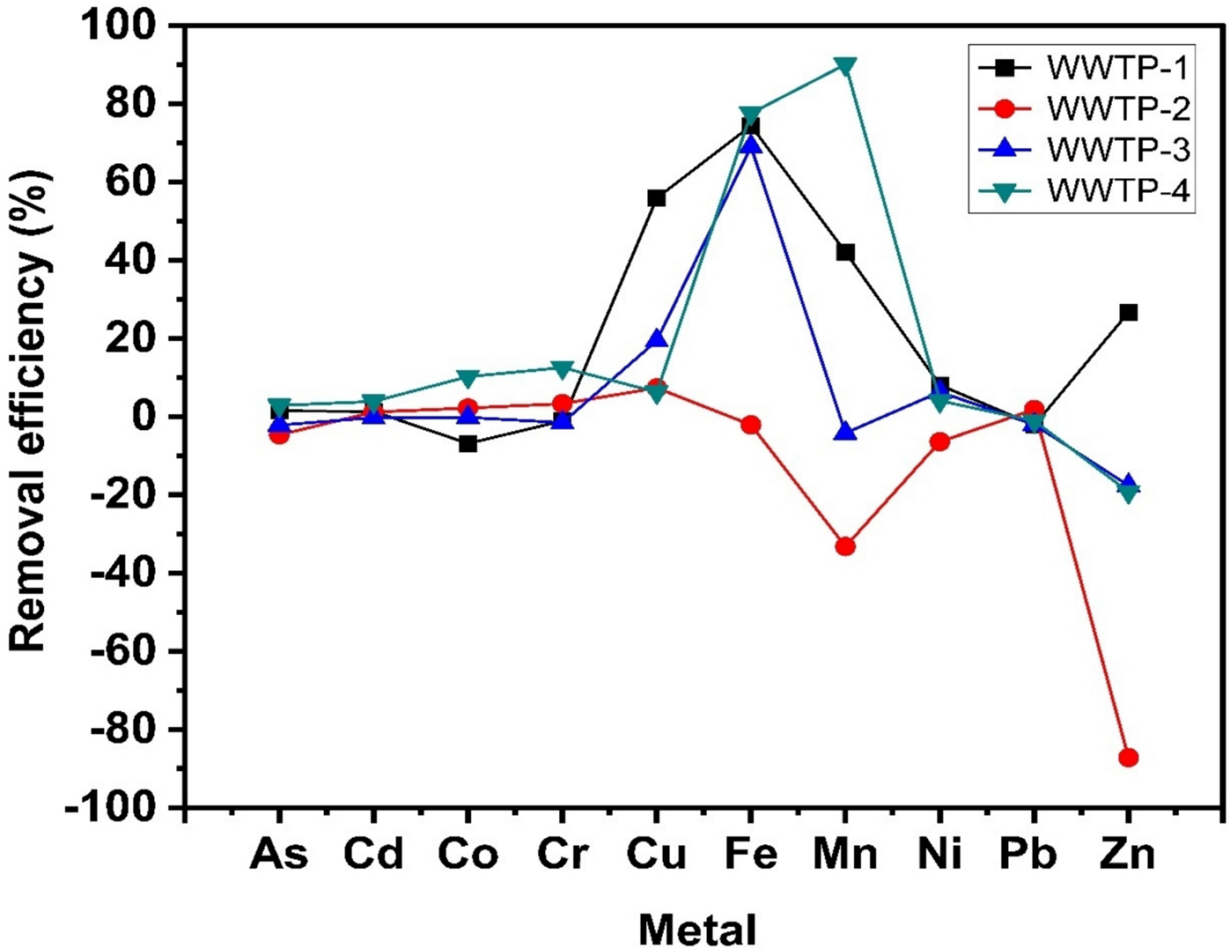
Removal efficiencies of heavy and trace metal species from wastewater in four WWTPs

Conventional WWTPs are not technically equipped for the removal of many organic and inorganic contaminants of environmental and human health concern. This is primarily because these were designed for the treatment of domestic water with the main focus on nutrients, suspended solids, biodegradable organics, pathogens, volatile organics and odours (Cantinho et al., 2016). Compared to their counterpart organic contaminants, metals are characteristically difficult to remove because they are non-volatile and non-biodegradable. However, adsorption onto suspended solids, bioaccumulation and biosorption may contribute to the removal of some metals from the wastewater during treatment (Karvelas et al., 2003). Ultimately these may account for reduced metal concentrations in the final treated effluents. To estimate the concentrations of metals removed during treatment in the four WWTPs, the metal removal efficiency percentages were calculated (Fig. 4). The results show high variability in the removal of metals between the four WWTPs and among the different metals. For metals such as As, Cd and Pb, the removal percentages were very low with maximum removal percentages of 2.87, 2.29 and 3.92% respectively. Metals that were removed to some extent in at least 2 WWTPs were Co, Cr, Cu, Fe, Mn and Ni. At WWTP-1 the highest removal percentage was recorded for Fe at 74.41%, and 5 other metals were removed in the order Fe > Cu > Mn > Zn > Ni > As. At WWWTP-2 Cu recorded the highest removal efficiency at 7.33% and for the other metals the removal efficiency was less than 5% (Cr > Co > Pb > Cd >). At WWWTP-3 Fe recorded the highest removal percentage at 69.02%. Other metals that were removed were Cu (19.47%) and Ni (6.33%). WWTP-4 recorded the highest number of metals with significant removal efficiency (Mn > Fe > Cr > Co > Cu > Ni > Cd > As) with Mn recording the highest removal percentage (90.11%). For metals such Zn (WWTPs 2, 3 and 4), and Mn in (WWTP-2), concentrations were significantly higher in effluents than influents. This means that these metals were enriched during the treatment process. The enrichment of metallic elements is a common process in WWTPs and occurs when insoluble metal hydroxides in sludge are resolubilized back into the wastewater as influenced by the metal uptake kinetics, metal solubility, and water pH (Moloi et al., 2020). A similar trend was previously observed at the Phuthaditjhaba (Ni, Mg, As) and Harrismith (Fe and Co) WWTPs located in Durban (Moloi et al., 2020). Similarly in three Vaal WWTPs As was enriched (Nyamukamba et al., 2019) whilst at Thohoyandou WWTP, Cu, Fe, Mn and Zn were enriched (Edokpayi et al., 2015).

A comparison of metal removal efficiencies reported in the present study to those previously reported locally confirms the high variability in metal removal efficiency (Table S4). For most metals the reported metal removal efficiencies fall within the range of the data for all the SA WWTPs, although some (Cd, Cr and Zn) are on the lower end of the spectrum. For Mn the percentage removal (90.11%) recorded for WWTP-4 is the highest among all the WWTPs. Available literature has shown that metal removal efficiency in WWTPs is not only affected by influent concentrations, but also the physicochemical properties of the wastewater as well as treatment processes applied (Edokpayi et al., 2015). Notably, the influent metal concentrations in the four WWTPs under study are higher than those reported in the other plants which could explain the inefficiency in metal removal. Furthermore, the four WWTPs under study are categorized as conventional and use activated sludge at the secondary treatment stage. The activated sludge process is inherently not efficient in the removal of metallic elements. Hence advanced WWTPs incorporate advanced treatment technologies such as ion exchange, adsorption and membrane technology to improve the efficiency of metal removal in WWTPs (Rathi et al., 2021). However, WWTPs in South Africa face myriad of the most fundamental challenges such that upgrading the systems is almost non feasible. Currently a more feasible alternative would be to minimize the amounts of contaminants reaching the WWTPs through catchment management.

Failure to significantly remove metals in WWTPs raises environmental and human health concerns as the effluents discharged into river systems may lead to deterioration of water quality. In all the studied WWTPs effluent concentrations of As, Cd, Co, Cr, Cu, Fe, Mn, Pb and Zn exceeded the permissible thresholds set by the Department of Water Affairs (National Water Act, 1998). High metal concentrations exceeding the set guidelines have previously been reported in Durban WWTPs (Fe and Mn), Eastern Cape (Cd), Limpopo (Zn, Cr, Cu, Mn, and Pb), and in 3 Vaal WWTPs (As, Cr, Cu, Fe, Mn, and Zn) (Edokpayi et al., 2015; Moloi et al., 2020; Nyamukamba et al., 2019; Olujimi et al., 2016). This is a cause for concern because inefficiently treated WWTP effluent pose a potential hazard to receiving resource environments. This confirms numerous reports to the effect that SA water treatment services are collapsing primarily because of deteriorating infrastructure, lack of skilled labour, system overload, vandalism of infrastructure, and the general poor performance of municipalities (Ntombela et al., 2016; Oberholster & Ashton, 2008; Department of Water and Sanitation, 2022).

### Metal concentrations in surface water

To establish the possible contribution of WWTPs on the heavy and trace metal loads of the four rivers into which effluents are discharged, metal concentrations were evaluated at two points before (upstream) and after (downstream) effluent discharge. The results reveal an indiscriminate distribution of metals in surface water before and after discharge of WWTP effluent (Table 4). As noted for the influent and effluent samples, As, Cu, Fe, Mn, Ni and Zn were detected in high abundance in surface water. Fe was detected as the most abundant metal in both the upstream and downstream samples with the concentrations ranging between 0.69—4.81 mg L^−1^ (upstream) and 1.25—4.63 mg L^−1^ (downstream). The high abundance of Fe is not unusual as it is a naturally abundant metal in the earth crust hence normal natural deposits are not uncommon. Furthermore, a range of anthropogenic sources such as mining, eroding roofing sheets, steel and iron cookware and crockery, tyre wear, industries (metal surface treatment, electroplating, electronics, organic chemicals, steel manufacturing) contribute to the high environmental concentrations (Masindi et al., 2021). Exceedingly high concentrations of Fe were previously reported from the Plankenburg (48 mg L^−1^) and the Diep (513 mg L^−1^) Rivers in the Western Cape (Jackson et al., 2009). Nonetheless lower concentrations have also been previously reported in some rivers around SA including Meultspan Lake (below detection limit to 2.08 mg L^−1^), Coalplex (0.28—0.52 mg L^−1^), Natref (0.12—0.50 mg L^−1^) (Mollo et al., 2022), Mvudi river (0.70—2.65 mg L^−1^) (Edokpayi et al., 2016).

**Table 4:** Metal concentrations (mg L^−1^) in surface water collected from four river systems receiving wastewater effluent discharge

Metal	RS-1		RS-2	RS-3		RS-4		*Permissible values	
	upstream	downstream	downstream	upstream	downstream	upstream	downstream	agricultural use	domestic use
As	0.350 ± 0.024	0.366 ± 0.034	0.383 ± 0.056	0.419 ± 0.094	0.419 ± 0.083	0.537 ± 0.197	0.459 ± 0.032	0.1	0.01
Cd	0.139 ± 0.006	0.140 ± 0.005	0.139 ± 0.005	0.139 ± 0.003	0.140 ± 0.003	0.140 ± 0.003	0.138 ± 0.003	0.01	0.005
Co	0.131 ± 0.005	0.132 ± 0.005	0.186 ± 0.059	0.134 ± 0.006	0.140 ± 0.003	0.323 ± 0.143	0.222 ± 0.076	0.05	N/A
Cr	0.127 ± 0.005	0.129 ± 0.007	0.132 ± 0.011	0.126 ± 0.005	0.129 ± 0.006	0.129 ± 0.007	0.128 ± 0.006	0.1	0.05
Cu	0.191 ± 0.047	0.216 ± 0.088	0.235 ± 0.116	0.237 ± 0.102	0.335 ± 0.140	0.224 ± 0.077	0.161 ± 0.028	0.2	10
Fe	1.200 ± 0.204	1.248 ± 0.079	4.631 ± 0.527	0.687 ± 0.002	1.893 ± 0.108	4.811 ± 0.658	3.282 ± 0.054	5	0.1
Mn	0.150 ± 0.000	0.725 ± 0.146	2.137 ± 0.939	0.147 ± 0.026	1.729 ± 0.041	0.921 ± 0.395	0.628 ± 0.033	0.05	0.05
Ni	0.141 ± 0.015	0.153 ± 0.030	0.246 ± 0.122	0.186 ± 0.058	0.169 ± 0.034	0.441 ± 0.239	0.287 ± 0.136	0.2	N/A
Pb	0.185 ± 0.012	0.188 ± 0.014	0.187 ± 0.013	0.187 ± 0.011	0.190 ± 0.009	0.187 ± 0.008	0.187 ± 0.010	0.2	0.01
Zn	0.246 ± 0.109	0.615 ± 0.019	0.641 ± 0.025	0.414 ± 0.079	0.583 ± 0.036	1.131 ± 0.029	0.323 ± 0.183	N/A	3

*Guidelines set by DWAF** (**Department of Water Affairs and Forestry, 1996b; Department of Water Affairs and Forestry, 1996a)

At RS-1 upstream metal concentrations ranged between 0.127 and 1.199 mg L^−1^ and the order of decreasing abundance was Fe > As > Zn > Cu > Pb > Mn > Ni > Cd > Co > Cr. The downstream metal concentrations ranged between 0.129 and 1.247 mg L^−1^ and metal abundance decreased in the order Fe > Mn > Zn > As > Cu > Pb > Ni > Cd > Co > Cr. In RS-2 downstream concentrations ranged between 0.132 and 4.631 mg L^−1^ and the order of decreasing abundance was Fe > Mn > Zn > As > Ni > Cu > Pb > Co > Cd > Cr. For RS-3 upstream metal concentrations were slightly lower than the other streams as they ranged between 0.125 and 0.680 mg L^−1^ with the concentrations decreasing in the other Fe > As > Zn > Cu > Pb > Ni > Mn > Cd > Co > Cr. Comparatively the downstream metal concentrations ranged between 0.128 and 1.893 mg L^−1^ and the order of decreasing abundance was Fe > Mn > Zn > As > Cu > Pb > Ni > Co > Cd > Cr. In RS-4 upstream metal concentrations ranged between 0.129 and 4.811 mg L^−1^ and decreased in the order Fe > Zn > Mn > As > Ni > Co > Cu > Pb > Cd > Cr. Similarly, the most abundant metal downstream was Fe (3.282 mg L^−1^) and the least abundant was Cr (0.127 mg L^−1^). Metal concentrations decreased in the order: Mn > Fe > As > Zn > Ni > Co > Pb > Cu > Cd > Cr. A comparison of the metal concentrations reported in this work with those of local rivers available in literature (Table S5) shows generally elevated metal levels in the Gauteng rivers. For example, the highest concentrations of As, Cd, Co, Mn and Pb were recorded for Gauteng Rivers.

The occurrence of metals in the river water at points before WWTP effluent discharge suggest that natural sources as well as anthropogenic activities (agriculture, domestic activities, mining) within the rivers’ catchment contribute significantly to metal loads. In RS-4 downstream concentrations of Co (p = 0.035), Cr (p = 0.026), Cu (p = 0.050), Cd (p = 0.021), and Ni (p = 0.036) were significantly lower than the upstream concentrations. This implies negligible contribution of WWTP effluent on the metal loads and underscores the significant contributions from uncontrolled waste management, inflow of domestic sewage and agriculture. However, for some metals downstream concentrations were higher than the upstream concentrations, implying possible enrichment by WWTP effluents. For example, in RS-1 downstream concentrations of As (p = 0.031), Co (p = 0.002) and Pb (p = 0.026) were significantly higher than the upstream values. Similarly in RS-3 downstream concentrations of Co (p = 0.016), Cr (p = 0.005), Cu (p = 0.008), Cd (p = 0.004), Fe (p = 0.017), Mn (p = 0.032) and Zn (p = 0.005) were significantly higher than upstream concentrations. This observation corroborates reports on the significant contribution of WWTP effluents on surface water metal loads. A trend previously noted at the Wilge River where the Harrismith WWTP contributes 50% of the metal loads. The Phuthaditjhaba WWTP as well enriches Pb, As, Cu, Cr, Ni, and Zn concentrations at the Elands River (Moloi et al., 2020).

Some of the metal concentrations reported in the current study exceeded the permissible levels set for safe domestic and agricultural uses. These metals could cause detrimental effects to aquatic life and the human beings who use surface water for domestic and agricultural purposes. As, Cd and Mn upstream and downstream concentrations in all 4 the river systems exceeded both the agriculture and domestic water use guideline set by the DWA. On the other hand, Cr, Fe and Pb values exceeded the domestic use guideline. Whilst, Co, and some Cu and Ni concentrations were in excess of the agriculture guideline (Department of Water Affairs & Forestry, 1996b). High metal concentrations in excess of the stipulated values have previously been reported in SA rivers including Nzhelele River in Limpopo (Cr, Fe, Mn, Pb and Zn), Steelport River (Cr, Cu, Mn, Zn), Plankenburg (Cu, Zn, Fe), Diep River (Cu, Fe, Mn, Zn) and Mvudi River (Cr, Fe, Mn, Pb) (Edokpayi et al., 2017; Addo-Bediako et al., 2018; Edokpayi et al., 2016).

## Human health risk assessment

Metals present in surface water and sludge carry significant risks to individuals using both sludge and surface water. Therefore, it remains crucial to quantitatively estimate the potential human health risks associated with exposure to these metals. In human beings’ exposure to metals occurs through various pathways including ingestion, inhalation and dermal absorption through skin contact.

### Human health risk assessment—surface water

Human health risks associated with the exposure to metals in water through ingestion and dermal contact were estimated for surface water at the downstream points using the indices ADD, HQ, HI, CR and RI. Comparing oral and dermal routes of exposure, ADD values for dermal contact were higher (Table 5) implying that in the four river systems dermal contact to metals is the primary route of exposure. This contradicts previous observations by (Bakare & Adeyinka 2022) who reported that oral ingestion presented more risks. Between the four river systems the ADD values decreased in the order RS-2 > RS-4 > RS-3 > RS-1. In all four river systems the highest ADD values were estimated for Fe, Mn and Zn. This likely relates to the high concentrations reported for these metals in surface water. Nonetheless, in all the four rivers, oral and dermal ADD values for As, Cd, and Cr exceeded the corresponding Minimal Risk Level (MRL) values derived by the Agency for Toxic Substances and Disease Registry (ATSDR) (Agency for Toxic Substances and Disease Registry (ATSDR), 2025). This implies that chronic exposure to the surface water could lead to appreciable adverse health effects.

**Table 5:** Average daily dose calculated to estimate average daily intake of metals through ingestion and dermal contact of surface water collected from river systems receiving effluent discharge from WWTP-1, WWTP-2, WWTP-3, WWTP-4

Metal	ADD (mg/kg bw/day)												MRL (mg/kg bw/day)
	RS-1			RS-2			RS-3			RS-4			
	ADD_ingest_	ADD_dermal_	ADD	ADD_ingest_	ADD_dermal_	ADD	ADD_ingest_	ADD_dermal_	ADD	ADD_ingest_	ADD_dermal_	ADD	
As	**0.011**	**0.020**	**0.032**	**0.012**	**0.021**	**0.033**	**0.013**	**0.023**	**0.036**	**0.014**	**0.026**	**0.040**	0.0003
Cd	**0.001**	**0.002**	**0.003**	**0.004**	**0.008**	**0.012**	**0.004**	**0.008**	**0.012**	**0.004**	**0.008**	**0.012**	0.0001
Co	0.004	0.003	0.007	0.006	0.004	0.010	0.004	0.003	0.007	0.007	0.005	0.012	0.02
Cr	**0.004**	**0.014**	**0.018**	**0.004**	**0.015**	**0.019**	**0.004**	**0.014**	**0.018**	**0.004**	**0.014**	**0.018**	0.0009
Cu	0.007	0.012	0.019	0.007	0.013	0.020	0.010	0.019	0.029	0.005	0.009	0.014	0.02
Fe	0.039	0.210	0.248	0.144	0.778	0.922	0.059	0.318	0.377	0.102	0.551	0.653	N/A
Mn	0.023	0.041	0.063	0.066	0.120	0.186	0.054	0.097	0.151	0.020	0.035	0.055	0.16
Ni	0.005	0.002	0.006	0.008	0.003	0.010	0.005	0.002	0.007	0.009	0.003	0.012	N/A
Pb	0.006	0.001	0.007	0.006	0.001	0.007	0.006	0.001	0.007	0.006	0.001	0.007	N/A
Zn	0.019	0.021	0.040	0.020	0.022	0.041	0.018	0.020	0.038	0.010	0.011	0.021	0.03
**ADD**	**0.444**			**1.261**			**0.683**			**0.844**			

*ADD values above the corresponding MRL are written in bold

The HQ_ingest_ and HQ_dermal_ as well as THQ (Table 6) values for individual metals were calculated to estimate non carcinogenic risks associated with ingestion and dermal contact to surface water. Furthermore, RI values were calculated for each river system. Except for Cd, the HQ_dermal_ values were significantly higher than the HQ_ingest_ values implying that exposure to contaminated water through bathing, swimming or performing spiritual rituals carries more risks compared to oral ingestion. For As, Cd, Cr, and Pb both HQ_ingest_ and HQ_dermal_ values were greater than the threshold value of one, in all four sampling points. On the other hand, for Cu (RS-1, RS-2 and RS-3) and Mn (all four river systems) only HQ _dermal_ values were above one. Among the studied metals THQ values for As, Cd, Cr, Cu, Mn and Pb were greater than the threshold value of one in all four rivers. While the THQ value for Ni was above unity only in RS-4. All calculated HI values were above the threshold value of one. These imply that in the long-term individuals who use water from the four river systems through ingestion, bathing, swimming, and religious purposes are likely to develop non-carcinogenic health effects. Notably Cr, As and Mn were the major contributors to the high HI values in all four river systems. This contradicts previous works in which As was identified as the main contributor (Bakare &Adeyinka 2022; Moloi et al., 2020).

**Table 6:** Non carcinogenic risk assessment for metal exposure in adults through ingestion and dermal contact of surface water collected from river systems RS-1, RS-2, RS-3 and RS-4 receiving effluent discharge from WWTP-1, WWTP-2, WWTP-3, WWTP-4 respectively

Metal	*HQ											
	RS-1			RS-2			RS-3			RS–4		
	HQ_ingest_	HQ_dermal_	THQ	HQ_ingest_	HQ_dermal_	THQ	HQ_ingest_	HQ_dermal_	THQ	HQ_ingest_	HQ_dermal_	THQ
As	**37.93**	**68.281**	**106.215**	**39.696**	**71.453**	**111.148**	**43.427**	**78.169**	**121.596**	**47.573**	**85.631**	**133.204**
Cd	**1.06**	**3.806**	**4.863**	**4.322**	**15.559**	**19.881**	**4.353**	**15.671**	**20.024**	**4.291**	**15.447**	**19.738**
Co	0.205	0.185	0.390	0.289	0.260	0.549	0.218	0.196	0.414	0.345	0.311	0.656
Cr	**1.337**	**240.662**	**241.999**	**1.368**	**246.259**	**247.627**	**1.337**	**240.662**	**241.999**	**1.327**	**238.797**	**240.124**
Cu	0.168	**1.007**	**1.175**	0.183	**1.096**	**1.279**	0.260	**1.562**	**1.823**	0.125	0.751	0.876
Fe	0.055	N/A	0.55	0.206	N/A	0.206	0.084	N/A	0.084	0.146	N/A	0.146
Mn	0.161	**22.053**	**22.214**	0.475	**65.002**	**65.477**	0.384	**52.592**	**52.976**	0.139	**19.102**	**19.242**
Ni	0.238	0.317	0.555	0.382	0.510	0.892	0.263	0.350	0.613	0.446	0.595	**1.041**
Pb	**1.670**	**2.104**	**3.775**	**1.661**	**2.093**	**3.754**	**1.688**	**2.127**	**3.815**	**1.661**	**2.093**	**3.754**
Zn	0.064	0.344	0.408	0.066	0.359	0.425	0.060	0.326	0.387	0.033	0.181	0.214
*HI	**381.649**			**451.239**			**443.730**			**418.995**		

*HQ and HI values above 1 are written in bold

Carcinogenic risk (CR) was calculated considering oral ingestion of As, Cd, Cr, Ni and Pb and dermal contact of Ni and Pb. Subsequently RI values were commuted for individual river systems **(**Table 7**)**. Except for Pb, all calculated CR values were above the threshold value of 1.0 × 10^–3^ indicative of a high possibility of developing adverse carcinogenic health effects. Across all sites, RI values were in the range 1.41 × 10^–1^ to 2.30 × 10^–1^. These values are indicative of very high risks of cancer development for individuals exposed to the surface water either through oral ingestion or dermal contact.

**Table 7:** Carcinogenic risk factor assessment for metal exposure via water consumption and dermal contact in river systems RS-1, RS-2, RS-3 and RS-4 receiving effluent discharge from WWTP-1, WWTP-2, WWTP-3, WWTP-4 respectively

Metal	CR											
	RS-1			RS-2			RS-3			RS-4		
	CR ingest	CR dermal	TCR	CR ingest	CR dermal	TCR	CR ingest	CR dermal	TCR	CR ingest	CR dermal	TCR
As	1.7 × 10^–2^	-	1.7 × 10^–2^	1.8 × 10^–2^	-	1.8 × 10^–2^	2.0 × 10^–2^	-	2.0 × 10^–2^	2.1 × 10^–2^	-	2.1 × 10^–2^
Cd	1.0 × 10^–3^	-	1.0 × 10^–3^	2.0 × 10^–3^	-	2.0 × 10^–3^	2.0 × 10^–3^	-	2.0 × 10^–3^	2.0 × 10^–3^	-	2.0 × 10^–3^
Cr	2.0 × 10^–3^	-	2.0 × 10^–3^	2.0 × 10^–3^	-	2.0 × 10^–3^	2.0 × 10^–3^	-	2.0 × 10^–3^	2.0 × 10^–3^	-	2.0 × 10^–3^
Ni	8.0 × 10^–3^	3.4 × 10^–2^	4.2 × 10^–2^	1.3 × 10^–2^	5.5 × 10^–2^	6.8 × 10^–2^	9.0 × 10^–3^	3.8 × 10^–2^	4.7 × 10^–2^	1.5 × 10^–2^	6.4 × 10^–2^	7.9 × 10^–2^
Pb	4.97 × 10^–5^	4.5 × 10^–2^	4.5 × 10^–2^	4.9 × 10^–5^	4.4 × 10^–2^	4.5 × 10^–2^	5.0 × 10^–5^	4.5 × 10^–2^	4.5 × 10^–2^	4.9 × 10^–5^	4.4 × 10^–2^	4.5 × 10^–2^
**RI**	**1.41 × 10**^**–1**^			**2.30 × 10**^**–1**^			**1.96 × 10**^**–1**^			**1.79 × 10**^**–1**^		

### Human health risk assessment—sludge

Workers handling sewage sludge during treatment in WWTPs as well as application in agricultural fields are ultimately the most exposed to contaminants present in the sludge. Therefore, the HHRA was conducted for adult workers considering incidental ingestion, dermal contact and inhalation of the anaerobically digested sludge. To estimate the average daily exposure ADD_ingest_, ADD_dermal_ and ADD_inhale_ values estimated from the mean concentrations reported for each metal are presented in Table S6. The cumulative ADD values are presented in Table 8. When comparing the three routes of exposure, ADD_ingest_ values were highest followed by ADD_dermal_ and ADD_inhale_ the least. In fact, ADD_inhale_ values were 3–4 orders of magnitude lower than ADD_ingest_ and ADD_dermal_, hence these may be considered negligible. This implies that for sludge, the primary route of exposure is ingestion followed by dermal contact. This is in line with previous reports in which incidental ingestion was identified as the primary route of exposure to metals present in sludge (Tytła & Widziewicz‑Rzońca, 2023; Rocha et al., 2025). When considering the different metals, for all the exposure routes the highest ADD values were recorded for Zn, Ni, Mn, Pb and Cu. For all the studied metals the ADD values were below the RfD values signifying that the average daily doses are within the guideline limits that adults can tolerate.

**Table 8:** Cumulative Average daily dose (ADD) calculated to estimate metal exposure via sludge ingestion, dermal contact and inhalation

Metal	ADD			
	WWTP-1	WWTP-2	WWTP-3	WWTP-4
Cd	8.17 × 10^–6^	1.46 × 10^–5^	1.28 × 10^–5^	1.01 × 10^–5^
Co	5.02 × 10^–5^	2.34 × 10^–4^	4.47 × 10^–5^	2.31 × 10^–4^
Cr	5.57 × 10^–5^	3.15 × 10^–4^	4.93 × 10^–4^	1.05 × 10^–4^
Cu	8.33 × 10^–5^	2.39 × 10^–5^	1.03 × 10^–4^	9.50 × 10^–5^
Fe	4.08 × 10^–5^	8.62 × 10^–5^	2.08 × 10^–5^	1.52 × 10^–4^
Mn	9.07 × 10^–5^	7.91 × 10^–4^	8.42 × 10^–4^	4.19 × 10^–4^
Ni	2.34 × 10^–4^	2.93 × 10^–4^	4.44 × 10^–4^	1.68 × 10^–4^
Pb	6.35 × 10^–5^	4.12 × 10^–5^	9.48 × 10^–5^	1.03 × 10^–4^
Zn	1.10 × 10^–3^	8.05 × 10^–4^	8.14 × 10^–4^	5.98 × 10^–4^
**Total**	**1.73 × 10**^**–3**^	**2.60 × 10**^**–3**^	**3.0 × 10**^**–3**^	**1.88 × 10**^**–3**^

The calculated HQ (Table S7) and THQ values (Table 9) for all the metals under study were below the threshold value of 1. This means that potential non carcinogenic risks emanating from sludge ingestion, dermal contact and inhalation were negligible. Regarding the assessment of carcinogenic risks, computed CR_ingest_, CR_dermal_ and CR_inhale_ values for for Cd, Cr, Ni, Pb and Zn are shown in Table S8 while the corresponding TCR values in Table 9. The TCR values show negligible (Pb, Zn), low (Cd), medium (Cr) and medium to high (Ni) carcinogenic risks. However, RI values indicate medium (WWTP-1), high (WWTP-2 and WWTP-4) and extremely high (WWTP-3) carcinogenic risks emanating from exposure to all five metals present in sludge. Therefore, there is a possibility that individuals handling sludge could develop cancer in the long run due to exposure to metals present in sludge. This underscores the need for reinforced precautionary measures to minimise exposure to sludge.

**Table 9:** Non carcinogenic and carcinogenic risk factor assessment for metal exposure via sludge consumption**,** dermal contact and inhalation

Metal	THQ				TCR			
	WWTP-1	WWTP-2	WWTP-3	WWTP-4	WWTP-1	WWTP-2	WWTP-3	WWTP-4
Cd	8.20 × 10^–3^	1.47 × 10^–2^	1.29 × 10^–2^	1.06 × 10^–2^	4.07 × 10^–6^	7.28 × 10^–6^	6.39 × 10^–6^	5.04 × 10^–6^
Co	2.51 × 10^–3^	1.17 × 10^–2^	2.23 × 10^–3^	1.15 × 10^–2^	-	-	-	-
Cr	2.16 × 10^–2^	1.22 × 10^–1^	1.92 × 10^–1^	4.06 × 10^–2^	2.78 × 10^–5^	1.57 × 10^–4^	2.46 × 10^–4^	5.22 × 10^–5^
Cu	2.10 × 10^–3^	6.02 × 10^–4^	2.59 × 10^–3^	2.39 × 10^–3^	-	-	-	-
Fe	6.0 × 10^–5^	1.23 × 10^–4^	2.96 × 10^–5^	2.17 × 10^–4^	-	-	-	-
Mn	8.1 × 10^–4^	7.09 × 10^–3^	7.54 × 10^–3^	3.76 × 10^–3^	-	-	-	-
Ni	1.18 × 10^–2^	1.48 × 10^–2^	2.24 × 10^–2^	8.50 × 10^–3^	3.97 × 10^–4^	4.96 × 10^–4^	7.53 × 10^–4^	2.85 × 10^–4^
Pb	2.03 × 10^–2^	1.32 × 10^–2^	3.03 × 10^–2^	3.31 × 10^–2^	5.32 × 10^–7^	3.44 × 10^–7^	7.97 × 10^–7^	8.70 × 10^–7^
Zn	4.59 × 10^–3^	3.37 × 10^–3^	3.40 × 10^–3^	2.50 × 10^–3^	6.36 × 10^–14^	4.65 × 10^–14^	4.71 × 10^–14^	3.46 × 10^–14^
**HI**	**7.21 × 10**^**–2**^	**1.88 × 10**^**–1**^	**2.73 × 10**^**–1**^	**1.13 × 10**^**–1**^	**-**	**-**	**-**	**-**
**RI**	**-**	**-**	**-**	**-**	**4.29 × 10**^**–4**^	**6.61 × 10**^**–4**^	**1.07 × 10**^**–3**^	**3.44 × 10**^**–4**^

## Conclusions

This study reports on the indiscriminate occurrence of metals in influents, effluents and sludge in four WWTPs as well as their receiving streams in the Gauteng Province. While physicochemical parameters of the WWTP effluents were within the range suitable for safe environmental discharge and agricultural use, concentrations of As, Cr and Pb exceeded the permissible values for safe river discharge. These high concentrations could limit the potential reuse of WWTP effluents in irrigation as well as pose human health and ecotoxicological risks in receiving streams. In the corresponding receiving streams, contributions of WWTPs to metal loads could not be confirmed with certainty in this study. The high metal concentrations in both the upstream and downstream points suggest substantial metal loading from anthropogenic activities along the course of the studied streams as possible sources. Concentrations for As, Cd, Co, Fe, Mn, Ni and Pb exceeded the threshold values for agricultural and or domestic use. Based on its physicochemical parameters, anaerobically digested sludge samples were suitable for applications in agriculture. However, the concentrations of Ni in WWTP-1, 2 and 3 downgraded the sludge to pollutant class c which is less suitable for use in agriculture. Human health risk assessment of surface water revealed a high possibility of noncarcinogenic and carcinogenic risks associated with the ingestion and dermal contact of surface water. These findings confirm that metal contamination in aquatic ecosystems is a threat to human health. Thus underscore the need to upgrade WWTPs to be able to remove metals as well as minimize metal leaching from anthropogenic activities into aquatic systems. Human health risk assessments for workers and farmers handling sludge revealed probable non-carcinogenic and carcinogenic risks emanating from incidental sludge ingestion. These highlight the need for proper protective gear to minimize exposure. Further research on the ecotoxicological risks of surface water as well as ecological risks emanating from sludge application in agriculture are crucial. Furthermore, quantifying metal concentrations in sediments could be significant in ascertaining the contribution of WWTP effluent towards metal contamination in the study area.

## Supplementary Information

Below is the link to the electronic supplementary material.

Supplementary file1 (DOCX 2072 KB)

## Data Availability

No datasets were generated or analysed during the current study.
