# Supplementary material for: Occurrence and health risk assessment of trace and heavy metals in four wastewater treatment plants and their receiving surface waters in Gauteng Province, South Africa
Source: Environ Monit Assess. 2026 Jul 20;198(8):862. doi: 10.1007/s10661-026-15686-3 (PMC13385251; doi:10.1007/s10661-026-15686-3)
Supplement: Supplementary file 1 — Supplementary file1 (DOCX 2072 KB) [file 10661_2026_15686_MOESM1_ESM.docx]

**Supplementary material for**

**Occurrence and health risk assessment of trace and heavy metals in four wastewater treatment plants and their receiving surface waters in Gauteng Province South Africa**

Lukhele Thabile^1^, Msagati Titus Alfred Makudali^1*^

^1^ Institute for Nanotechnology and Water Sustainability (iNanoWS), College of Science Engineering and Technology, University of South Africa, UNISA Science Campus, Johannesburg, South Africa

*Corresponding author

Email: [msagatam@unisa.ac.za](mailto:msagatam@unisa.ac.za)

[lukhelethabilen@yahoo.co.uk](mailto:lukhelethabilen@yahoo.co.uk)

**List of tables**

**Table S1:** Reference ingestion dose (RfD) and carcinogenic slope factors (CSF) of metals according to SA and USEPA guidelines

**Table S2:** Metal limits for South African sewage sludge (mg kg^-1^)

**Table S2:** Average daily dose (mg kg^-1^ bw day^-1^) of metals in adults due to ingestion, dermal contact and inhalation of anaerobically digested sludge collected from four WWTPs

**Table S3:** Comparison of metal concentrations in the 4 WWTPs and others from different parts of South Africa

**Table S4:** Comparison of metal removal efficiency (%) in the four Gauteng WWTPs against others from in South Africa

**Table S5**: Comparison of metal concentrations in the four Gauteng Rivers and others from different parts around South Africa

**Table S6:** Non carcinogenic (HQ) risk assessment for metal exposure in adults through ingestion, dermal contact and inhalation of anaerobically digested sludge collected from four WWTPs

**Table S7:** Carcinogenic risk factor assessment for metal exposure through ingestion, dermal contact and inhalation of anaerobically digested sludge collected from four WWTPs

**Table S8:** Carcinogenic risk factor assessment for metal exposure through ingestion, dermal contact and inhalation of anaerobically digested sludge collected from four WWTPs

**Table S1:** Reference ingestion dose (RfD) and carcinogenic slope factors (CSF) of metals according to SA and USEPA guidelines (Bakare & Adeyinka, 2022; Moloi et al., 2020).

| **Metal** | **Kp** (**cm h^-1^)** | **RfD_ingest_** | **RfD_dermal_** | **RfD_inhale_** | **CSF_ingest_** | **CSF_dermal_** | **CSF_inhale_** |
| --- | --- | --- | --- | --- | --- | --- | --- |
| **As** | 0.001 | 0.0003 | 0.0003 | 0.00001 | 1.5 |  | 6.3 |
| **Cd** | 0.001 | 0.001 | 0.00001 |  | 0.5 |  |  |
| **Co** | 0.0004 | 0.02 | 0.016 | 0.0000286 |  |  | 42 |
| **Cr** | 0.002 | 0.003 | 0.00006 | 0.0402 | 0.501 |  |  |
| **Cu** | 0.001 | 0.04 | 0.012 |  |  |  |  |
| **Fe** | 0.003 | 0.7 |  |  |  | 20 |  |
| **Mn** | 0.001 | 0.14 | 0.00184 | 0.0206 | 1.5 |  | 0.84 |
| **Ni** | 0.0002 | 0.02 | 0.0054 | 0.00352 | 1.7 | 20 |  |
| **Pb** | 0.0001 | 0.0035 | 0.0005 | 0.3 | 0.0085 | 42.5 | 0.42 |
| **Zn** | 0.0006 | 0.3 | 0.06 | 0.00001 |  |  | 6.3 |

**Table S2:** Metal limits for sewage sludge in south Africa (Snyman & Herselman, 2006)

| **Metal** | **Permissible values (mg kg^-1^)** | | |
| --- | --- | --- | --- |
|  | **Class A** | **Class B** | **Class C** |
| **As** | <40 | 40 - 75 | >75 |
| **Cd** | <40 | 40 - 85 | >85 |
| **Cr** | <1200 | 1200 - 3000 | >3000 |
| **Cu** | <1500 | 1500 - 4300 | >4300 |
| **Ni** | <420 | 420 | >420 |
| **Pb** | <300 | 300 - 840 | >840 |
| **Zn** | <2800 | 2800 - 7500 | >2800 |

**Table S3:** Comparison of metal concentrations in the 4 WWTPs and others from different parts of South Africa

|  |  | **As** | **Cd** | **Co** | **Cr** | **Cu** | **Fe** | **Mn** | **Ni** | **Pb** | **Zn** |
| --- | --- | --- | --- | --- | --- | --- | --- | --- | --- | --- | --- |
| **Durban**  **WWTPs** | **Influent** | 0.3 | 0.4 | 0.1 | 0.1 | - | 0.233 – 1.809 | 0.18 – 0.8 | 0.18 – 0.8 | 0.3 – 0.4 | 0.2 – 0.35 |
|  | **Effluent** | 0.3 | 0.4 | 0.1 | 0.1 | - | 0.2 – 0.8 | 0.18 – 0.5 | 0.18 – 0.2 | 0.29 – 0.3 | 0.1 – 0.35 |
| **Capetown and Stellenbosch** | **Influent** | 0.005 – 0.044 | 0.001 – 0.017 | 0.034 – 0.012 | 0.031 – 0.22 | 0.021 – 1.189 | - | - | 0.008 – 0.78 | 0.01 – 0.061 | 0.4 – 5.13 |
|  | **Effluent** | 0.001 – 0.006 | 0.001 – 0.003 | 0.0002 – 0.005 | 0.024 – 0.153 | 0.007 – 0.055 | - | - | 0.008 – 0.159 | 0.001 – 0.011 | - |
| **Thohoyandou WWTP** | **Influent** | - | - | - | 0.030 – 0.476 | 0.017 – 0.055 | 0.74 – 1.37 | 0.098 – 0.303 | - | 0.002 – 0.072 | 0.071 – 0.776 |
|  | **Effluent** | - | - | - | 0.030 – 0.422 | 0.021 – 0.267 | 0.49 – 1.33 | 0.042 – 0.899 | - | 0.008 – 0.042 | 0.046 – 0.217 |
| **Vaal WWTPs** | **Influent** | 0.053 – 0.065 | <0.01 | 0.265 – 0.324 | <0.020 | 0.191 – 0.208 | 0.132 – 0.396 | 0.064 – 0.249 | 0.323 – 2.271 | <0.01 | 1.121 – 2.437 |
|  | **Effluent** | 0.060 – 0.075 | <0.01 | 0.239 – 0.305 | <0.020 | 0.054 – 0.152 | 0.105 – 0.267 | 0.022 – 0.032 | 0.030 – 0.268 | <0.01 | 0.706 – 0.810 |
| **Durban**  **WWTPs** | **Influent** | 0.0003 – 0.0012 | 0.0001 | 0.00058 – 0.00059 | 0.0025 – 0.005 | 0.003 – 0.02 | 0.330 - 0.632 | 0.181 – 0.275 | 0.0025 - 0.0032 | 0.0028 - 0.0041 | 0.01 – 0.18 |
|  | **Effluent** | 0.0004 - 0.0012 | 0.00003 – 0.0001 | 0.0005 - 0.002 | 0.0002 – 0.002 | 0.0003 – 0.0013 | 0.112 – 0.725 | 0.089 – 0.143 | 0.002 - 0.003 | - | 0.002 – 0.060 |
| **Gauteng WWTPs (This study)** | **Influent** | 0.368 – 0.401 | 0.140 – 0.144 | 0.136 – 0.153 | 0.133 – 0.150 | 0.191 – 0.405 | 1.668 – 4.813 | 0.581- 1.512 | 0.155 – 0.231 | 0.183 – 0.191 | 0.185 – 0.191 |
|  | **Effluent** | 0.363 – 0.391 | 0.138 – 0.140 | 0.137 – 0.188 | 0.132 – 0.138 | 0.178 – 0.310 | 0.374 – 4.914 | 0.145 – 2.014 | 0.142 – 0.246 | 0.185 – 0.193 | 0.437 – 1.100 |

References: Bakare and Adenyika (2022); Olujimi et al., 2016; Edokpayi et al., 2015; Agoro et al., 2020; Nyamukamba et al., 2022; Moloi et al 2020

* Not measured

**Table S4:** Comparison of metal removal efficiency (%) in the four Gauteng WWTPs against others from in South Africa

| **Metal** | **Percentage removal efficiency** | | | | | | | | |
| --- | --- | --- | --- | --- | --- | --- | --- | --- | --- |
|  | **WWTP-1** | **WWTP-2** | **WWTP-3** | **WWTP-4** | **Durban WWTPs** | **Eastern Cape WWTPs** | **Vaal WWTPs** | **Capetown and Stellenbosch WWTPs** | **Thohoyandou WWTP** |
| **As** | 1.45 | -4.64 | -2.15 | 2.87 | -60 - 48 | - | -41.5 – (-10.8) | 22.14 -94.12 | - |
| **Cd** | 1.38 | 1.20 | -0.23 | 3.92 | 20 - 50 | 0 | - | 2.38 – 92.33 | - |
| **Co** | -6.83 | 2.24 | -0.16 | 10.25 | -50 - 5 | - | 5.9 – 9.9 | 5.79 – 88.89 | - |
| **Cr** | -1.05 | 3.36 | -1.51 | 12.54 | 15 - 50 | - | - | 3.86 – 83.11 | 0 - 31 |
| **Cu** | 55.94 | 7.33 | 19.47 | 6.29 | 80 - 93 | 0 – 23.4 | 20.8 – 74.0 | 15.83 – 98.10 | -21.9 - 36 |
| **Fe** | 74.41 | -2.10 | 69.07 | 77.58 | -39 - 70 | 34.7 – 86.6 | 20.5 – 47.4 | - | -20 - 35 |
| **Mn** | 42.09 | -33.24 | -4.17 | 90.11 | 13 - 55 | - | 65.6 – 87.1 | - | -62.3 - 86 |
| **Ni** | 8.12 | -6.39 | 6.35 | 4.05 | -10 - 20 | - | 6.2 – 89.1 | 1.5 – 91.5 | - |
| **Pb** | -2.15 | 1.84 | -2.05 | -1.07 | 20 - 40 | - | - | 19.7 – 92.6 | 0 - 42 |
| **Zn** | 26.73 | -87.19 | -17.62 | -19.24 | 68 - 70 | - | 21.4 – 71.0 | 6.7 – 84.5 | -9.6 - 35 |
| **Reference** | This study | | | | Moloi (2022) | Agoro (2020) | Nyamukamba (2019) | Olujimi (2016) | Edokpayi (2015) |

**Table S5**: Comparison of metal concentrations in the four Gauteng Rivers and others from different parts around South Africa

| **Metal** | **Concentration mg L^-1^** | | | | | | | | | | |
| --- | --- | --- | --- | --- | --- | --- | --- | --- | --- | --- | --- |
|  | **Plankenburg** | **Diep** | **Nzhelele** | **Mvudi** | **Steelport** | **Elands** | **Wilge** | **Meulsteenpan** | **Coalplex** | **Natref** | **Gauteng** |
| **As** | - | - | - | - | - | ND | ND | 6.2x10^-4^ – 0.004 | 0.004 – 0.010 | 0.002 – 0.011 | 0.366 – 0.537 |
| **Cd** | - | ^-^ | 4x10^-4^ – 2x10^-3^ | 3x10^-4^ – 2x10^-3^ | - | 0.01 | ND | 3.6x10^-4^ – 0.001 | 6x10^-5^ – 2.1x10^-4^ | 7x10^-5^ – 7.7x10^-4^ | 0.138 – 0.140 |
| **Co** | - | - | - | - | - | - | - | 0.003 – 0.009 | 0.002 – 0.108 | 0.0019 – 0.0021 | 0.131 – 0.222 |
| **Cr** | - | - | 0.045 – 0.396 | 0.015 – 0.357 | 0.02 – 0.06 | ND | ND | 0.002 – 0.004 | 3x10^-4^ – 0.001 | 0.005 – 0.060 | 0.126 – 0.129 |
| **Cu** | 0.30 – 2.2 | 0.1 – 0.8 | 0.026 – 0.066 | 0.024 – 0.185 | 0.01 – 0.012 | 0.02 | ND | 0.004 – 0.008 | 0.002 – 0.005 | 0.006 – 0.007 | 0.161 – 0.335 |
| **Fe** | 0.3 – 48 | 0.1 - 513 | 1.028 – 4.991 | 0.702 – 2.645 | - | 0.580 | 1.42 | ND – 2.08 | 0.052 – 0.272 | 0.005 – 0.115 | 0.687 – 4.811 |
| **Mn** | 0.00 – 0.4 | 0 – 1.3 | 0.052 – 0.5 | 0.081 – 0.521 | 0.027 – 0.66 | 0.180 | 0.590 | 0.011 – 0.568 | 0.035 – 0.272 | 0.041 – 0.060 | 0.147 – 2.137 |
| **Ni** | 0.1 – 0.5 | 0 – 0.4 | - | - | 0.01 – 0.02 | ND | ND | 9x10^-5^ – 6.8x10^-4^ | ND | ND – 6.7x10^-4^ | 0.141 – 0.441 |
| **Pb** | ND | ND | 0.001 – 0.013 | 0.002 – 0.042 | - | - |  | - | 8.7x10^-4^ – 0.0038 | 0.002 - 0.003 | 0.185 – 0.190 |
| **Zn** | 0.0 – 0.1 | 0.1 – 4.4 | 0.042 – 0.131 | - | 0.02 – 0.07 | 0.02 | 0.02 | - | - | - | 0.246 – 1.131 |
| **Reference** | Jackson et al 2009 | | Edkopayi et al., (2015) | Edkopayi et al (2016) | Addo-Bediako et al., (2018) | Moloi et al 2020 | | Mollo et al (2022) | | | This Study |

ND = below detection limit

**Table S6:** Average daily dose (mg kg^-1^ bw day^-1^) of metals in adults due to ingestion, dermal contact and inhalation of anaerobically digested sludge collected from four WWTPs

| **Metal** | **WWTP-1** | | | **WWTP-2** | | | **WWTP-3** | | | **WWTP-4** | | |
| --- | --- | --- | --- | --- | --- | --- | --- | --- | --- | --- | --- | --- |
|  | **Add_ingest_** | **ADD_dermal_** | **ADD_inhale_** | **Add_ingest_** | **ADD_dermal_** | **ADD_inhale_** | **Add_ingest_** | **ADD_dermal_** | **ADD_inhale_** | **Add_ingest_** | **ADD_dermal_** | **ADD_inhale_** |
| **Cd** | 8.15x10^-6^ | 2.77x10^-8^ | 1.20x10^-15^ | 1.46x10^-5^ | 4.94x10^-8^ | 2.14x10^-15^ | 1.28x10^-5^ | 4.34x10^-8^ | 1.88x10^-15^ | 1.01x10^-5^ | 3.42x10^-8^ | 1.48x10^-15^ |
| **Co** | 5.01x10^-5^ | 1.7010^-7^ | 7.36x10^-15^ | 2.33x10^-4^ | 7.91x10^-7^ | 3.43x10^-14^ | 4.45x10^-5^ | 1.51x10^-7^ | 6.54x10^-15^ | 2.30x10^-4^ | 7.81x10^-7^ | 3.38x10^-14^ |
| **Cr** | 5.55x10^-5^ | 1.88x10^-7^ | 8.16x10^-15^ | 3.14x10^-4^ | 1.07x10^-6^ | 4.61x10^-14^ | 4.92x10^-4^ | 1.67x10^-6^ | 7.23x10^-14^ | 1.04x10^-4^ | 3.54x10^-7^ | 1.53x10^-14^ |
| **Cu** | 8.30x10^-5^ | 2.82x10^-7^ | 1.22x10^-14^ | 2.38x10^-5^ | 8.09x10^-8^ | 3.5 x10^-15^ | 1.02x10^-4^ | 3.48x10^-7^ | 1.51x10^-14^ | 9.47x10^-5^ | 3.22x10^-7^ | 1.39x10^-14^ |
| **Fe** | 4.07x10^-5^ | 1.38x10^-7^ | 5.98x10^-15^ | 8.59x10^-5^ | 2.92x10^-7^ | 1.26x10^-14^ | 2.07x10^-5^ | 7.04x10^-8^ | 3.05x10^-15^ | 1.52x10^-4^ | 5.15x10^-7^ | 2.23x10^-14^ |
| **Mn** | 9.04x10^-5^ | 3.07x10^-7^ | 1.33x10^-14^ | 7.89x10^-4^ | 2.68x10^-6^ | 1.16x10^-13^ | 8.39x10^-4^ | 2.85x10^-6^ | 1.23x10^-13^ | 4.18x10^-4^ | 1.42x10^-6^ | 6.15x10^-14^ |
| **Ni** | 2.34x10^-4^ | 7.93x10^-7^ | 3.43x10^-14^ | 2.92x10^-4^ | 9.9x10^-7^ | 4.29x10^-14^ | 4.43x10^-4^ | 1.50x10-^6^ | 6.51x10^-14^ | 1.68x10^-4^ | 5.70x10^-7^ | 2.47x10^-14^ |
| **Pb** | 6.22x10^-5^ | 1.27x10^-6^ | 9.15x10^-15^ | 4.04x10^-5^ | 8.2xX10^-7^ | 5.95x10^-15^ | 9.29x10^-5^ | 1.89x10^-6^ | 1.36x10^-14^ | 1.01x10^-4^ | 2.06x10^-6^ | 1.49x10^-14^ |
| **Zn** | 1.03x10^-3^ | 6.99x10^-5^ | 1.51x10^-13^ | 7.54x10^-4^ | 5.12x10^-5^ | 1.11x10^-13^ | 7.62x10^-4^ | 5.18x10^-5^ | 1.12x10^-13^ | 5.60x10^-4^ | 3.80x10^-5^ | 8.23x10^-14^ |
| **Total** | **1.65x10^-3^** | **7.30x10^-5^** | **2.43x10^-13^** | **3.0x10^-3^** | **5.79x10^-5^** | **3.74x10^-13^** | **3.0x10^-3^** | **6.03x10^-5^** | **4.13x10^-13^** | **2.0x10^-3^** | **4.41x10^-5^** | **3.0x10^-12^** |

**Table S7:** Non carcinogenic (HQ) risk assessment for metal exposure in adults through ingestion, dermal contact and inhalation of anaerobically digested sludge collected from four WWTPs

| **Metal** | **WWTP-1** | | | **WWTP-2** | | | **WWTP-3** | | | **WWTP-4** | | |
| --- | --- | --- | --- | --- | --- | --- | --- | --- | --- | --- | --- | --- |
|  | **HQ_ingest_** | **HQ_dermal_** | **HQ_inhale_** | **HQ_ingest_** | **HQ_dermal_** | **HQ_inhale_** | **HQ_ingest_** | **HQ_dermal_** | **HQ_inhale_** | **HQ_ingest_** | **HQ_dermal_** | **HQ_inhale_** |
| **Cd** | 8.0x10^-3^ | 6.0x10^-5^ | 1.20x10^-10^ | 1.46x10^-2^ | 9.89x10^-5^ | 2.14x10^-10^ | 1.28x10^-2^ | 8.67x10^-5^ | 1.88x10^-10^ | 1.01x10^-2^ | 6.85x10^-5^ | 1.48x10^-10^ |
| **Co** | 3.0x10^-3^ | 1.0x10^-5^ |  | 1.16x10^-2^ | 4.0x10^-5^ | - | 2.23x10^-3^ | 9.44x10^-6^ |  | 1.15x10^-2^ | 4.88x10^-5^ |  |
| **Cr** | 1.9x10^-2^ | 3.14x10^-3^ | 2.85x10^-10^ | 1.05x10^-1^ | 1.77x10^-2^ | 1.61x10^-9^ | 1.64x10^-1^ | 2.78x10^-2^ | 2.53x10^-9^ | 3.47x10^-2^ | 5.90x10^-3^ | 5.36x10^-10^ |
| **Cu** | 2.0x10^-3^ | 2.0x10^-5^ | 3.04x10^-13^ | 5.95x10^-4^ | 6.74x10^-6^ | 8.71x10^-14^ | 2.57x10^-3^ | 2.90x10^-10^ | 3.80x10^-13^ | 2.37x10^-3^ | 2.68x10^-5^ | 3.46x10^-10^ |
| **Fe** | - | - | - | 1.23x10^-4^ | - | - | 2.96x10^-5^ | - | - | 2.17x10^-4^ | - | - |
| **Mn** | 1x10^-3^ | 1.7x10^-4^ | - | 5.63x10^-3^ | 1.46x10^-3^ | - | 5.99x10^-3^ | 1.55x10^-3^ |  | 2.99x10^-3^ | 7.71x10^-4^ | - |
| **Ni** | 1.2 x10^-2^ | 1.5x10^-4^ | 1.67x10^-12^ | 1.46x10^-2^ | 1.83x10^-4^ | 2.08 x10^-12^ | 2.21x10^-2^ | 2.78x10^-4^ | 3.16x10^-12^ | 8.39x10^-3^ | 1.06x10^-6^ | 1.20x10^-10^ |
| **Pb** | 1.8x10^-2^ | 2.54x10^-4^ | 2.60x10^-12^ | 1.16x10^-2^ | 1.65x10^-3^ | 1.69 x10^-12^ | 2.65x10^-2^ | 3.78x10^-3^ | 3.88x10^-12^ | 2.90x10^-2^ | 4.13x10^-3^ | 4.23x10^-10^ |
| **Zn** | 3.0x10^-3^ | 1.16x10^-4^ | 5.04x10^-13^ | 2.51x10^-3^ | 8.53x10^-4^ | 3.69 x10^-13^ | 2.54x10^-3^ | 8.63x10^-4^ | 3.7 x10^-13^ | 1.87x10^-3^ | 6.33x10^-4^ | 2.74x10^-10^ |
| **Total** | **6.50x10^-2^** | **7.24x10^-3^** | **4.10x10^-10^** | **1.66x10^-1^** | **2.2x10^-2^** | **1.83 x10^-9^** | **2.39x10^-1^** | **3.44x10^-2^** | **2.72x10^-9^** | **1.01x10^-1^** | **1.2x10^-2^** | **6.9x10^-10^** |

**Table S8:** Carcinogenic risk factor assessment for metal exposure through ingestion, dermal contact and inhalation of anaerobically digested sludge collected from four WWTPs

| **Metal** | **WWTP-1** | | | **WWTP-2** | | | **WWTP-3** | | | **WWTP-4** | | |
| --- | --- | --- | --- | --- | --- | --- | --- | --- | --- | --- | --- | --- |
|  | **CR_ingest_** | **CR _dermal_** | **CR_inhale_** | **CR_ingest_** | **CR _dermal_** | **CR_inhale_** | **CR_ingest_** | **CR _dermal_** | **CR_inhale_** | **CR_ingest_** | **CR _dermal_** | **CR_inhale_** |
| **Cd** | 4.07x10^-6^ | 1.53x10^-12^ | 7.55x10^-15^ | 7.28x10^-6^ | 4.89x10^-12^ | 1.35x10^-14^ | 6.39x10^-6^ | 3.76x10^-12^ | 1.18x10^-14^ | 5.04x10^-6^ | 2.34x10^-12^ | 9.34x10^-15^ |
| **Cr** | 2.78x10^-5^ | 5.92x10^-10^ | 3.43x10^-13^ | 1.57x10^-4^ | 1.89x10^-8^ | 1.94x10^-12^ | 2.46x10^-4^ | 4.65 x10^-8^ | 3.04x10^-12^ | 5.22x10^-5^ | 2.09x10^-9^ | 6.44x10^-13^ |
| **Ni** | 3.97x10^-4^ | 1.16x10^-10^ | 2.88x10^-14^ | 4.96x10^-4^ | 1.81x10^-10^ | 3.60x10^-14^ | 7.53x10^-4^ | 4.18x10^-10^ | 5.47x10^-14^ | 2.85x10^-4^ | 6.02x10^-11^ | 2.07x10^-14^ |
| **Pb** | 5.29x10^-7^ | 3.21x10^-3^ | - | 3.44x10^-7^ | 1.36x10^-9^ | - | 7.90x10^-7^ | 7.16x10^-9^ | - | 8.61x10^-7^ | 8.52x10^-9^ | - |
| **Zn** | - | - | 6.36x10^-14^ | - | - | 4.66x10^-13^ | - | - | 4.71x10^-14^ | - | - | 3.46x10^-14^ |
| **Total** | **4.29x10^-4^** | **3.92x10^-9^** | **4.43x10^-13^** | **6.61x10^-4^** | **2.05x10^-8^** | **2.0x10^-11^** | **1.0x10^-3^** | **5.0X10^-8^** | **3.15x10^-12^** | **3.43x10^-4^** | **1.07x10^-7^** | **7.0x10^-12^** |

**References**

Addo-Badiako, A., Matlou, K., & Makushu, E. (2018). Heavy metal concentrations in water and sediment of the Steelpoort River, Olifants River System, South Africa. *African Journal of Aquatic Science* (Vol. 43, Issue 4, pp. 413–416). <https://doi.org/10.2989/16085914.2018.1524745>

Agoro, M. A., Adeniji, A. O., Adefisoye, M. A., & Okoh, O. O. (2020). Heavy metals in wastewater and sewage sludge from selected municipal treatment plants in eastern cape province, South Africa. *Water (Switzerland)*, *12*(10). <https://doi.org/10.3390/w12102746>

Bakare, B. F., & Adeyinka, G. C. (2022). Evaluating the Potential Health Risks of Selected Heavy Metals across Four Wastewater Treatment Water Works in Durban, South Africa. *Toxics*, *10*(6). <https://doi.org/10.3390/toxics10060340>

Edokpayi, J. N., Odiyo, J. O., Msagati, T. A. M., & Popoola, E. O. (2015). Removal Efficiency of Faecal Indicator Organisms, Nutrients and Heavy Metals from a Peri-Urban Wastewater Treatment Plant in Thohoyandou, Limpopo Province, South Africa. *International Journal of Environmental Research and Public Health*, *12*(7), 7300–7320. <https://doi.org/10.3390/ijerph120707300>

Edokpayi, J. N., Odiyo, J. O., Popoola, E. O., & Msagati, T. A. M. (2017). Evaluation of temporary seasonal variation of heavy metals and their potential ecological risk in Nzhelele River, South Africa. *Open Chemistry*, *15*(1), 272–282. <https://doi.org/10.1515/chem-2017-0033>

Edokpayi, J. N., Odiyo, J. O., Popoola, O. E., & Msagati, T. A. M. (2016). Assessment of trace metals contamination of surface water and sediment: A case study of Mvudi River, South Africa. *Sustainability (Switzerland)*, *8*(2). <https://doi.org/10.3390/su8020135>

Jackson, V. A., Paulse, A. N., Odendaal, J. P., & Khan, W. (2009). Investigation into the metal contamination of the Plankenburg and Diep Rivers, Western Cape, South Africa. <http://www.wrc.org.za>

Mollo, V. M., Nomngongo, P. N., & Ramontja, J. (2022). Evaluation of Surface Water Quality Using Various Indices for Heavy Metals in Sasolburg, South Africa. *Water (Switzerland)*, *14*(15). <https://doi.org/10.3390/w14152375>

Moloi, M., Ozekeke, O., & Patricks, V.O. (2020). Probabilistic health risk assessment of heavy metals at wastewater discharge points within the Vaal basin, South Africa. *International journal of hygiene and environmental health*, 224 (2020) 113421. [https://doi.org/10.1016/j.ijheh.2019.113421](https://doi.org/10.3390/w14152375)

Nyamukamba, P., Moloto, M. J., Tavengwa, N., & Ejidike, I. P. (2019). Evaluating physicochemical parameters, heavy metals, and antibiotics in the influents and final effluents of South African wastewater treatment plants. *Polish Journal of Environmental Studies*, *28*(3), 1305–1312. <https://doi.org/10.15244/pjoes/85122>

Olujimi, O. O., Fatoki, O. S., Daso, A. P., & Oputu. (2016). Variation in levels and removal efficiency of heavy and trace metals from wastewater treatment plant effluents in Cape Town and Stellenbosch, South Africa. African Journal of Biotechnology *15*(23), 1101–1135. <https://doi.org/10.5897/AJB12.1723>

Snyman, H., Herselman, J., 2006. Guidelines for the Utilization and Disposal of Wastewater Sludge, Volume 2: Requirements for the Agricultural Use of Wastewater Sludge. WRC Rep. TT 262/06. Water Research Commission, Pretoria, South Africa.
